# Comprehensive characterization and targeted treatment of a pediatric epithelioid glioblastoma with a rare TRIM24-NTRK2 fusion

**DOI:** 10.1038/s41698-025-01190-3

**Published:** 2025-12-02

**Authors:** Simon Keane, Ida Sjöberg, Lily Deland, Angelica Lindlöf, Thomas Olsson Bontell, Teresia Kling, Malin Blomstrand, Henrik Fagman, Jonas A. Nilsson, Daniel Nilsson, Magnus Sabel, Helena Carén, Frida Abel

**Affiliations:** 1 Clinical Genetics and Genomics, Department of Laboratory Medicine, Institute of Biomedicine, University of Gothenburg, Gothenburg, Sweden; 2 Sahlgrenska Centre for Cancer Research, Department of Medical Biochemistry and Cell Biology, Institute of Biomedicine, University of Gothenburg, Gothenburg, Sweden; 3 Department of Clinical Genetics and Genomics, Sahlgrenska University Hospital, Gothenburg, Sweden; 4 School of Bioscience, University of Skövde, Systems Biology Research Environment, University of Skövde, Skövde, Sweden; 5 Department of Physiology, Institute of Neuroscience and Physiology, Sahlgrenska Academy, University of Gothenburg, Gothenburg, Sweden; 6 Department of Clinical Pathology, Sahlgrenska University Hospital, Gothenburg, Sweden; 7 Department of Oncology, Institute of Clinical Sciences, Sahlgrenska Academy at the University of Gothenburg, Sahlgrenska University Hospital, Gothenburg, Sweden; 8 Department of Laboratory Medicine, Institute of Biomedicine, University of Gothenburg, Gothenburg, Sweden; 9 Sahlgrenska Center for Cancer Research, Department of Surgery, Institute of Clinical Sciences, Sahlgrenska Academy, University of Gothenburg, Gothenburg, Sweden; 10 Department of Neurosurgery, Sahlgrenska University Hospital, Gothenburg, Sweden; 11 Childhood Cancer Centre, Queen Silvia Children´s Hospital, Sahlgrenska University Hospital, Gothenburg, Sweden; 12 Department of Pediatrics, Institute of Clinical Sciences, Sahlgrenska Academy, University of Gothenburg, Gothenburg, Sweden

**Keywords:** CNS cancer, Molecular medicine, Translational research, Cancer genomics, Paediatric cancer

## Abstract

High-grade gliomas are one of the most lethal cancers and the leading cause of cancer-related mortality in children. Standard treatment typically involves resection, chemotherapy and radiation, yet offer limited improvement in survival rates. Targeted therapies are emerging to show effect in solid tumors harboring tyrosine-kinase activating fusion genes. Here, we present a thorough characterization of an epithelioid glioblastoma in a six-year-old patient, with four relapses and a variety of different treatment strategies. We identified a rare *TRIM24::NTRK2* fusion in the primary tumor, which enabled a targeted TRK-inhibitor treatment. Our findings show that the *TRIM24::NTRK2* fusion initially had oncogenic abilities, but this became less imperative as the tumor evolved. A general drug resistance to TRK-inhibition was documented. This study addresses the complex and adaptive nature of pediatric epithelioid glioblastomas and highlights the need for continued molecular profiling from relapses and various tumor regions to enable multitarget treatment approaches.

## Introduction

Central nervous system (CNS) tumors are the second most common childhood cancer, where pediatric high-grade glioma (pHGG) is the most aggressive and the leading cause of child mortality in high-income countries^1,2^. The reliance on molecular profiling is evident in the 2021 WHO CNS tumor classification, in which four main subtypes of pediatric diffuse high-grade gliomas are recognized, including: Diffuse midline glioma, H3K27-altered; Diffuse hemispheric glioma, H3G34-mutant; Diffuse pediatric-type high-grade glioma, H3-wild type and IDH-wild type; and Infant-type hemispheric glioma^3^. Other high-grade gliomas that rarely occur in pediatric patients are Epithelioid glioblastoma/Grade 3 pleomorphic xanthoastrocytoma and IDH-mutant astrocytomas^4^. These tumor types have associated clinical, genetic, and epigenetic features that are distinct from adult-type diffuse gliomas. Despite that, adult-type gliomas can occur in pediatric patients and vice versa^3^.

Standard treatment for pHGG in Sweden includes surgical resection with concurrent radiotherapy and chemotherapy, most commonly temozolomide combined with lomustine^5^. However, temozolomide has shown limited survival benefit in pediatric patients^6,7^. Moreover, treating HGG in children is significantly challenging due to the unique anatomical and physiological characteristics of the developing brain and the risk for long-term adverse treatment effects. Also, the presence of cancer stem cells (CSCs) can contribute to resistance and recurrence^8,9^.

Genomic profiling of pediatric tumors by whole genome sequencing (WGS) has recently been implemented in clinical routine in Sweden, identifying targetable precision-based molecular alterations in 26% of children^10^. Given the large degree of tumor heterogeneity in pHGG, diverse tumor drivers across patients, and the challenge with therapy-resistant CSCs^11,12^, a precision-guided treatment (PGT) approach is required throughout the disease course to improve patient outcomes and reduce treatment-related toxicities^13,14^. The main pathways activated in pHGGs are receptor tyrosine kinases (RTK), MAPK/ERK, and PI3K/AKT/mTOR through point mutations, gene fusions, or gene amplifications^15^. Fusion genes are attractive as both diagnostic markers and therapeutic targets, given their expression being restricted to tumor tissue. A recent study of patients with childhood cancer showed that PGT targeting fusions had a greater clinical benefit compared to targeting other alterations^16^. Aligned with emerging tumor-agnostic therapies, which focus on blocking the effects of specific signaling pathways promoting tumor growth, it is essential to identify the underlying tumor-driving genetic events. Several fusion genes have been identified in pHGG, most commonly in the infant type, involving kinases such as *ALK*, *MET*, *NTRK*, *ROS1*, and *FGFR*^15^. As sequencing technologies become more sophisticated, novel genetic alterations, including new fusion genes and partners, can be identified, advancing the development of precision care for pediatric gliomas^17,18^.

Here, we present a longitudinal case study of a pediatric patient diagnosed with epithelioid glioblastoma driven by a rare *TRIM24::NTRK2* fusion and concurrent homozygous *CDKN2A/B* deletion. By integrating molecular and clinical data throughout the patient´s disease course, this study enhances our understanding of cancer development and evaluates the efficacy of targeted therapeutic strategies.

## Results

### Case presentation

**Fig. 1:**
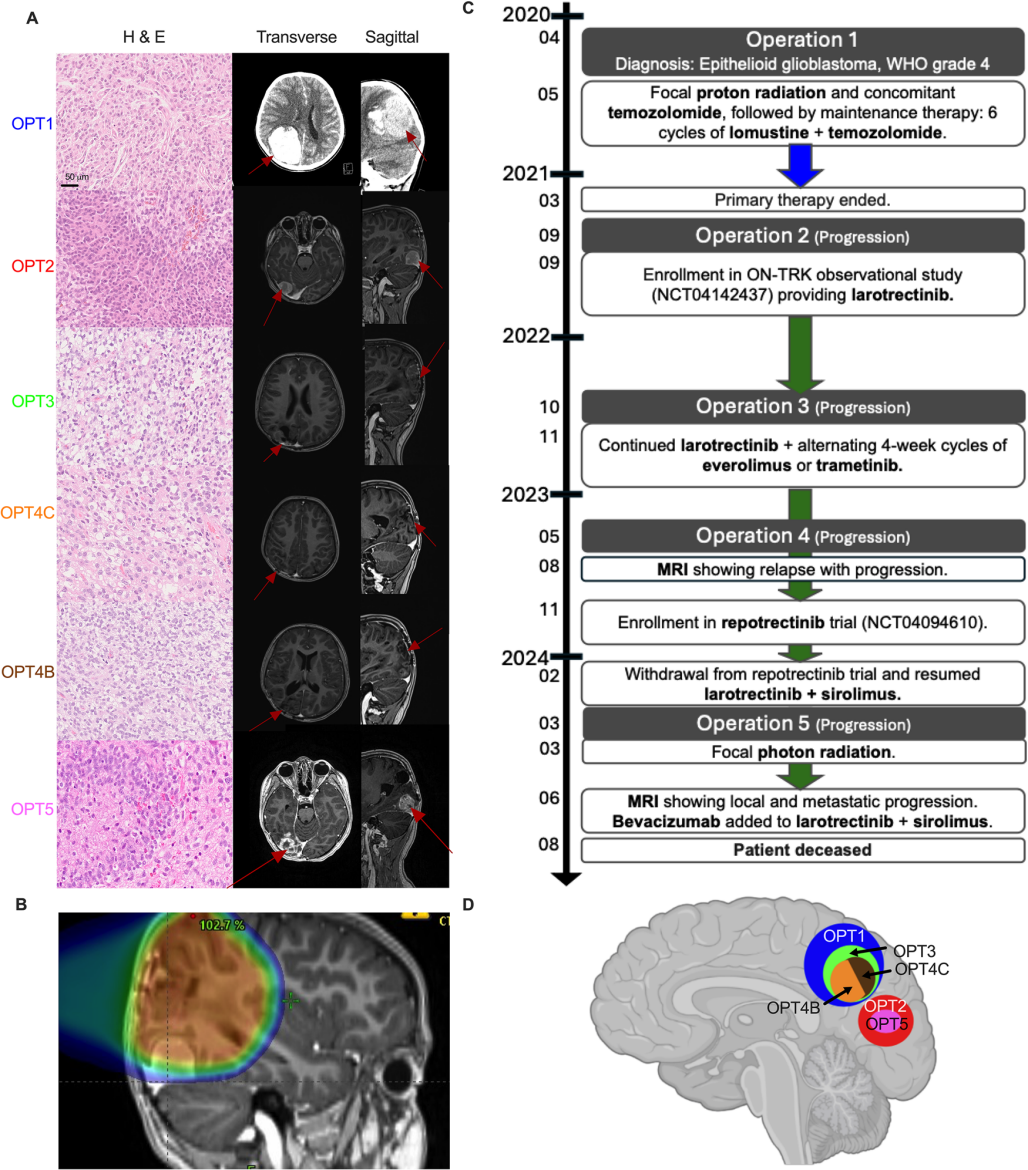
Patient history, MRI, and Histopathology of each operation. **A** Left: Hematoxylin and eoisin (H&E) stain from OPT1 showed a highly cellular astrocytic tumor with a high number of mitoses. Tumor tissue from OPT2 and OPT3 had similar morphology but higher cellularity and microvascular proliferation. OPT4C and OPT4B harbored cells with a clear cell morphology and high mitoses numbers. OPT5 was highly vascular with necrotic tissue. All six tumor specimens were classified as (anaplastic) PXA (diffuse glioma, MAPK activated, cell-cycle activated) by methylation array with calibrated scores over 0.9. Right: Preoperative CT (OPT1) and MRI (OPT2-5) with contrast in transverse and sagittal planes of the patient. **B** Matched MRI image from OPT2 and radiation plan from initial treatment indicating the irradiated brain area, with dosages ranging from 30% to 100% of full dose (59.4 Gy (RBE)). **C** Timeline of patient’s disease course including operations and treatment. **D** Schematic overview of location for the different tumors (Brain illustration created in BioRender. Deland, L. (2025) https://BioRender.com/zftx4oc).

In April 2020, a 6-year-old girl presented with a 6-month history of progressive headaches and vomiting, culminating in decreased consciousness on the day of presentation. An acute CT-scan revealed a tumor located in the right parietal lobe with an intratumoral hemorrhage. The tumor was completely resected (OPT1), and no evidence of further tumor dissemination in the CNS was observed. The tumor was difficult to diagnose, exhibiting GFAP and SOX10 negative and MAP2 positive. Routine clinical H&E-staining showed a highly cellular astrocytic tumor with an average of 6.5 mitoses/10 HPF (Ki-67 5–10%) and cells with prominent nucleoli and eosinophilic cytoplasm (Fig. 1A). Immunohistochemistry (IHC) showed negative staining of IDH1 R132H and BRAF V600E, INI1 was preserved, and TP53 not accumulated in a significant fraction of tumor cells. No mutations in *BRAF*, *KRAS*, *NRAS*, or *EGFR* were detected. An external pathological review found no evidence of fusion genes (NanoString panel of 88 genes, including *ALK, ROS1, NTRK1/2/3*, and *BRAF*). The methylation-based classification suggested anaplastic pleomorphic xanthoastrocytoma (-like) (PXA, score 0.99, Classifier 12.5), no *MGMT* promotor methylation, and the derived CNV profile indicated a homozygous deletion (HD) of *CDKN2A/B*. The PXA methylation class represents an astrocytic tumor with varied histology, primarily that of PXA or anaplastic PXA interposed with epithelioid glioblastoma tumors^19^. The final diagnosis was epithelioid glioblastoma, WHO grade 4. The patient was treated with focal proton therapy with concurrent temozolomide, followed by six cycles of temozolomide and lomustine.

### Clinical molecular analysis

**Fig. 2:**
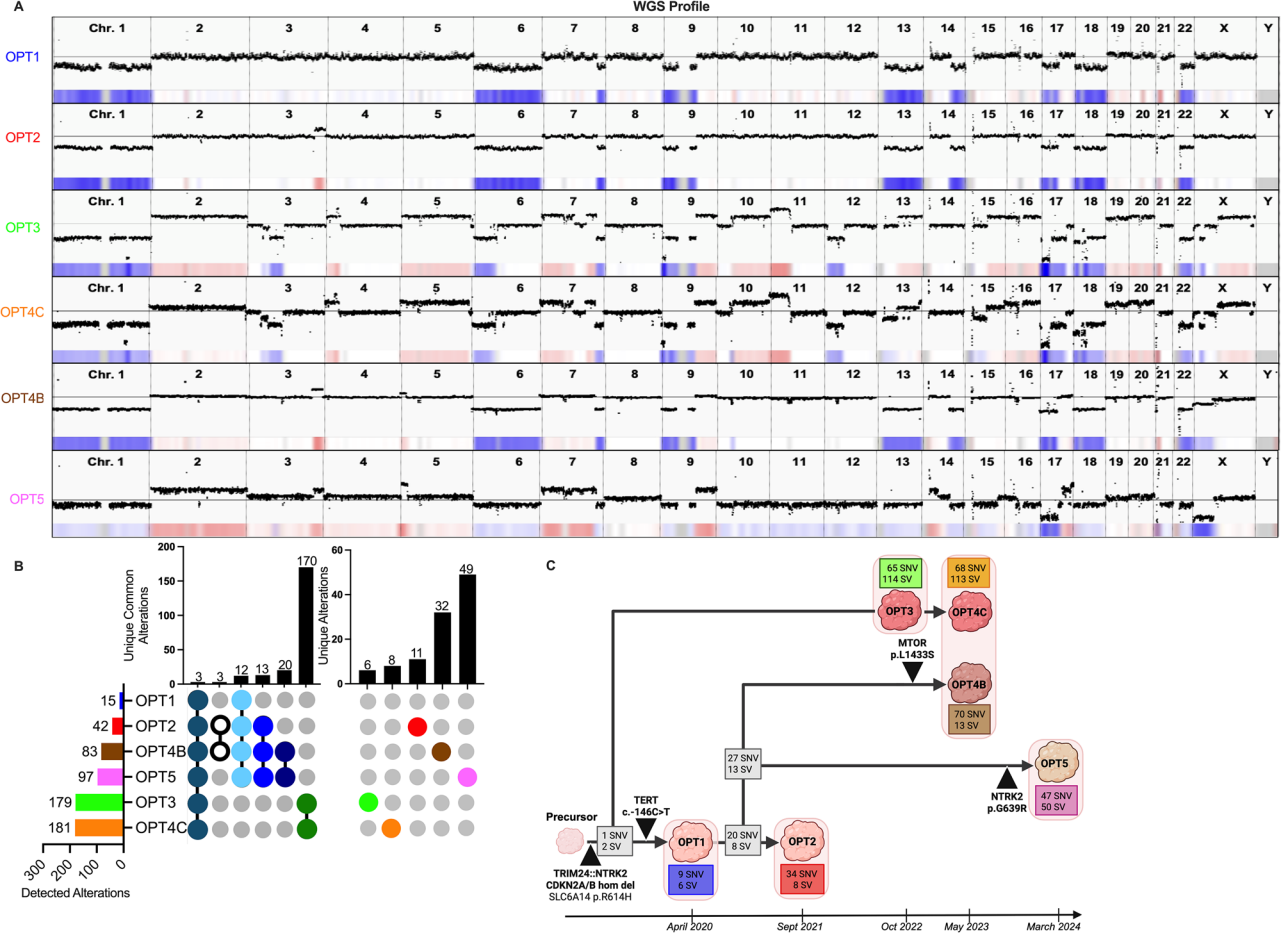
Genetic alterations and tumor development over time. **A** WGS CNV profile from operations 1 to 5 (OPT1-5) is displayed with a heatmap underneath indicating loss (blue) or gain (red). **B** Number of detected alterations (nonsynonymous (ns)SNVs/indels and all SVs) for each tumor specimen and shared common alterations between linkages and progressions. **C** Schematic tumor evolution model based on similarities and dissimilarities of genetic variants in the six tumor specimens and the five operation time points. The number of nsSNV/indel and SV alterations per branch and operation is shown within color-coded boxes. Key genetic events are labelled with red triangles. Created in BioRender. Deland, L. (2025) https://BioRender.com/zftx4oc.

WGS analysis of OPT1 showed several numerical and segmental chromosomal losses (Fig. 2A). Manta analysis revealed numerous structural variants (SVs), one in 9p21.3 confirming the *CDKN2A/B* HD, and one indicating a translocation between 7q33-34 and 9q21.32 resulting in a *TRIM24* (intron 13)-*NTRK2* (intron 12) fusion gene. The rearrangement was verified in tumor cells using FISH break-apart probe for *NTRK2*. RNA-sequencing revealed two in-frame *TRIM24::NTRK2* transcript isoforms; exon 12–14 and exon 12–13, both including an intact tyrosine kinase domain (TKD, Supplementary Fig. 1). Mutation analysis revealed nine somatic nonsynonymous (ns) single nucleotide variants (SNVs) of which one was classified as pathogenic/likely pathogenic (P/LP); a c.-146C > T *TERT* promoter mutation (p*TERT*; Table 1).

**Table 1:** Somatic Pathogenic (P) and Likely pathogenic (LP) variants in WGS and RNA-seq data

Tumor material	P/LP DNA variants	VAF	AMP Tier	TC (%) (Canvas)	TC (%) (histopathology)	Therapeutic regimen prior to relapse
OPT1, primary	**TRIM24::NTRK2 fusion**	14.%	IA	85%	approximately 80% (no mirrored material)	
	**CDKN2A/B homozygous deletion**	64%	IID			
	TERT c.-146 C > T	13%	III			
OPT2, relapse 1	**TRIM24::NTRK2 fusion**	31%	IA	91%	95% (mirrored material)	Focal proton radiation followed by lomustine and temozolomide
	**CDKN2A/B homozygous deletion**	84%	IID			
	TERT c.-146 C > T	53%	III			
OPT3, Relapse 2	**TRIM24::NTRK2 fusion**	37%	IA	88%	95% (mirrored material)	Larotrectinib
	**CDKN2A/B homozygous deletion**	84%	IID			
	RRM2B c.789+2 T > C	40%	III			
	RBFOX2::ITPK1 fusion	26%	III			
	MYO9A::RBFOX2 fusion	28%	III			
	ITPK1::MYO9A fusion	20%	III			
OPT4C (cranial), Relapse 3	**TRIM24::NTRK2 fusion**	33%	IA	52%	75% (mirrored material)	Larotrectinib with 4-week alternating cycles of everolimus or trametinib
	**CDKN2A/B homozygous deletion**	62%	IID			
	RRM2B c.789+2 T > C	27%	III			
	RBFOX2::ITPK1 fusion	14%	III			
	ITPK1::MYO9A fusion	12%	III			
OPT4B (basal), Relapse 3	**TRIM24::NTRK2 fusion**	35%	IA	92%	75% (mirrored material)	
	**MTOR p.L1433S**	35%	IIC			
	**CDKN2A/B homozygous deletion**	76%	IID			
	TERT c.-146C > T	61%	III			
	TYK2 p.P42fs*6	27%	III			
	GLB1 p.V439G	15%	III			
OPT5, Relapse 4	**TRIM24::NTRK2 fusion**	30%	IA	69%	85% (mirrored material)	Repotrectinib followed by larotrectinib and sirolimus
	**NTRK2 p.G639R**	17%	IC			
	**CDKN2A/B homozygous deletion**	60%	IID			
	TERT c.-146 C > T	61%	III			
	GLB1 p.V439G	57%	III			

Somatic Pathogenic (P) and Likely pathogenic (LP) variants in WGS and RNA-seq data from different tumor patient material according to QCI Interpret Tier classification, which is according to Association for Molecular Pathology (AMP) guidelines:^52^. Tier I; evidence level A: Variant of strong clinical significance; approved therapy, included in professional guidelines. Tier I; evidence level B: Variant of strong clinical significance; well-powered studies with consensus from experts in the field. Tier II; Evidence level C: Approved therapy for different tumor types or investigational therapies. Multiple small, published studies with some consensus. Tier II; evidence level D: Preclinical trials or a few case reports without consensus. Tier III; unclear clinical evidence level. Variants classified as AMP tiers I and II are shown in bold. VAF, Variant Allele Frequency. Tumor cell content (TC) was analyzed in mirrored/not mirrored FFPE tumor sections by a neuropathologist and calculated by the CNV calling tool Canvas.

Five months after initial treatment, a local recurrence was revealed with magnetic resonance imaging (MRI) in the right occipital lobe. The tumor was completely resected (OPT2) and showed similar morphology and genomic profile as OPT1 (Fig. 1A and Fig. 2A), with some additional mutations but no additional P/LP variants (Table 1 and Fig. 2B, C). The patient was considered for clinical trials with TRK-inhibitor and included in the ON-TRK observational study (NCT04142437), starting daily larotrectinib treatment, sustained for 12.5 months. However, another relapse in the right parietal lobe occurred in mid-2022 with subsequent resection (OPT3). WGS analysis showed a different copy number variation (CNV) profile with a large increase of SNVs and SVs compared to the two previous operations, and the p*TERT* mutation was lost (Table 1 and Fig. 2). After comprehensive review by medical professionals from multiple countries, it was decided to add 4-week-long alternating rotations of trametinib (MEK inhibitor) and everolimus (mTOR inhibitor) combined with continuous larotrectinib treatment.

In spring 2023, the patient again experienced a relapse with tumors growing from two distinct locations within the surgical cavity: cranial and basal. Complete resection was performed, and tissue was collected from both locations. While the cranial part (OPT4C) showed high genetic similarity to the OPT3 specimen, the basal part (OPT4B) was more similar to OPT1 and OPT2 (Fig. 2A). Mutation analysis of OPT4B showed multiple recurrent variants from OPT1/2 but also revealed a novel acquired MTOR p.L1433S resistance mutation. To explore new TRK-inhibitor regimens, the patient was enrolled in a repotrectinib clinical trial (NCT04094610). However, three months after commencement of treatment a relapse was observed in the occipital lobe and consequently repotrectinib was discontinued. Larotrectinib was resumed in combination with a novel mTOR-inhibitor; sirolimus. In March 2024, a complete resection of the tumor (OPT5) was performed. Histopathology displayed necrotic tissue, indicative of rapid growth (Ki-67 ~30% positivity), along with rich and proliferative tumor vascularization. CNV profiling and mutation analysis showed similarity to OPT1, OPT2, and OPT4B (Fig. 2A). While no MTOR mutation could be found in OPT5, the sequencing analysis revealed a new NTRK2 p.G639R resistance mutation in the TKD (Fig. 2D). The patient received larotrectinib and sirolimus treatment in conjunction with photon re-irradiation. MRI two months after radiotherapy revealed both local and metastatic progression. Despite this, the patient was still able to attend school, and a last treatment effort was made by adding bevacizumab (VEGF inhibitor) to larotrectinib and sirolimus. However, this was unsuccessful and the patient died from progressive disease two months later, more than four years after initial diagnosis.

### Genetic analyses reveal two distinct tumor lineages and significant differences between surgeries

CNV profiles revealed high similarities between OPT1, OPT2, OPT4B and OPT5, while OPT3 and OPT4C exhibited a derivative profile with additional CNVs, indicating two distinct tumor subclones (Fig. 2A). OPT3 and OPT4C also showed numerous nsSNV/indels and SVs (179 and 181, respectively), of which many were double stranded breaks compared to the other tumor samples (Fig. 2B, C). Generally, an increasing number of variants were observed over time, from operation 1 to 5; 15 alterations in OPT1, 42 in OPT2, 83 in OPT4B, and 97 in OPT5 (Fig. 2B, C). The OPT1 sample had three somatic alterations of clinical significance; *TRIM24::NTRK2*, *CDKN2A/B* HD, and *TERT* c.-146C > T (Table 1 and Fig. 2C). These variants were common to all tumor specimens except for the p*TERT* mutation which was lost in OPT3 and OP4C. In addition to *TRIM24::NTRK2* and *CDKN2A/B* HD, a *SLC6A14* missense variant (p.R614H) of unknown significance (VUS) was present in all six tumor specimens. Overall, OPT3 and OPT4C showed a unique mutation profile with several new VUS variants; *RRM2B* (c.789+2 T > C), *RBFOX::ITPK1, MYO9A::RBFOX2*, and *ITPK1::MYO9A* (Table 1). The findings were also confirmed in cultured OP1 and OP3 cells (Supplementary Fig. 2). OPT4B and OPT5 acquired additional pathogenic variants over time, including the resistance mutations *MTOR* p.L1433S and *NTRK2* p.G639R, respectively (Table 1).

### *TRIM24::NTRK2* displays oncogenic capacity

The biological effect of *TRIM24::NTRK2* on proliferation and oncogenic signaling was investigated in HEK293 cells. Increased cellular proliferation over 72 h was shown for both fusion transcript isoforms, exon 13 and exon 14, compared to the NTRK2^wt^ (Supplementary Fig. 3A). Activation of TRKB (pTRKb S516 targeting the activated state of the TKD) and phosphorylation of downstream oncogenic pathways; MAPK (pERK, pS6), PI3K (pAKT, pS6), and JAK/STAT (pSTAT) were quantified by western blot. A significant increased activation of pTRKB (6-fold) and mediators of all three pathways (pERK, pAKT, and pSTAT) was found in *TRIM24::NTRK2* transfected cells (Supplementary Fig. 3B). The highest activation was found in the PI3K pathway (pAKT, 2.9- to 3.3-fold) and the MAPK/PI3K downstream S6 mediator (pS6, 11.0- to 13.8-fold). No difference in oncogenic growth and signaling potential between the fusion isoforms was observed. The activation of oncogenic pathways was also confirmed by IHC in primary tumor tissue, showing the most prominent activation of MAPK pathway; pERK staining was elevated over time from operation 1-4, with the highest levels detected in OPT4C (Supplementary Fig. 4). The activation of PI3K (pAKT), on the other hand, remained relatively stable across all the samples. The pSTAT3 showed the most intense positive nuclear staining in OPT3 and OPT4B, indicating a shift to JAK/STAT-mediated signaling in later operations.

### Patient-derived cells show decreased response to TRK inhibitors over time in vitro

**Fig. 3:**
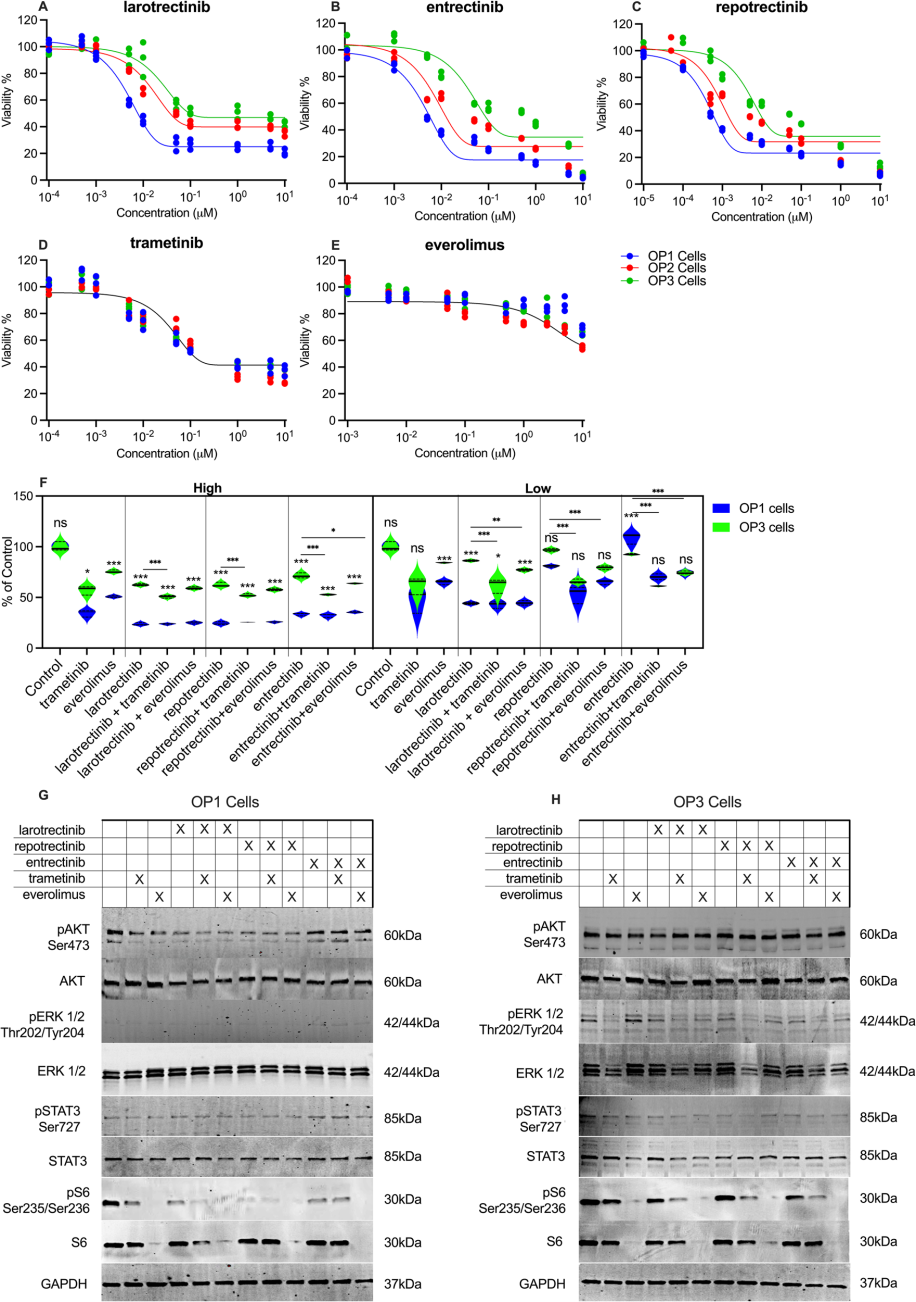
Dose-response curves for patient-derived tumor cells show acquired drug resistance lacking synergy between treatments. Dose-response curves for; **A** larotrectinib, **B** entrectinib, **C** repotrectinib, **D** trametinib, and **E** everolimus, across three different patient-derived primary cells, OP1 (blue), OP2 (red), and OP3 (green). The *y*-axis represents cell viability as a percentage, while the *x*-axis represents the drug concentration in micromolar (µM). A nonlinear curve of best fit was fitted to each of the datasets (black). **F** Single and combination drug treatment effect in OP1 and OP3 using a high (~IC50 of OP3) and a low (~IC50 of OP1) dose. The effect of the drug treatment on MAPK, PI3K, and JAK/STAT signaling pathways activation determined by western blot with phosphorylation status of pAKT (S473), pERK1/2 (T202/T204), pSTAT3 (S727), and pS6 (S235/S236). Data was normalized against total unphosphorylated protein levels for (**G**) OP1 and (**H**) OP3.

To assess whether the patient might benefit from another type of targeted treatment, three types of TRK-inhibitors (TRKi) were evaluated in patient-derived cell cultures from OP1, OP2, and OP3; larotrectinib (first generation TRKi, patients current/actual treatment at the time), entrectinib (first generation TRKi) and repotrectinib (second generation TRKi). Since the patients actual treatment also involved altering (4 week) cycles of the MEK inhibitor trametinib and the mTOR inhibitor everolimus, the efficacy of each TRKi in combination with trametinib and everolimus was also evaluated. The results showed that all three cell lines from different operations responded to all three TRKi (Fig. 3A–C) and trametinib (Fig. 3D) alone. Everolimus did not result in a clear dose-response relationship in any of the cell lines (Fig. 3E). Furthermore, the experiments showed decreased drug efficacy over time (from OP1 to OP3) for all three TRKi (Fig. 3A–C). Both entrectinib (*p* value > 0.01 for OP1 versus (vs) OP2 and > 0.001 for OP1 vs OP3) and larotrectinib (*p* value > 0.001 for OP1 vs OP2 and >0.001 for OP1 vs OP3) showed significant decreases in treatment efficacy over time. Whilst repotrectinib showed no difference in efficacy between OP1 and OP2 (*p* value > 0.05 for OP1 vs OP2), it showed a significant decreased anti-tumor effect in OP3 (*p* value > 0.01 for OP1 vs OP3). There was otherwise no significant difference in efficacy between the different TRKi drugs.

The efficacy of drug combinations showed no additive effects of trametinib or everolimus to any of the three TRKi at high doses in OP1 and OP3 (Fig. 3F). A Bliss Independence analysis showed significant antagonistic effects for all treatment combinations (Supplementary Table 4).

The effects of drug treatment on the phosphorylation status of pERK1/2, pAKT, pSTAT3, and pS6 was assessed in treated compared to untreated OP1 and OP3. Regardless of the drug and combinations, OP1 maintained a significantly lower level of pAKT and pERK across all treatments than OP3 (Fig. 3G, H). Larotrectinib and repotrectinib were highly efficient in depleting pAKT compared to entrectinib but showed no significant difference when combined with trametinib or everolimus. However, repotrectinib treatment showed the lowest degree of pS6 phosphorylation in OP1, but this effect was lost in OP3. Trametinib was less effective in OP3 than the equivalent treatment in OP1. STAT3 phosphorylation levels remained unchanged between OP1 and OP3.

### *TRIM24::NTRK2* fusion reveals diminished driver capacity in tumor relapses

**Fig. 4:**
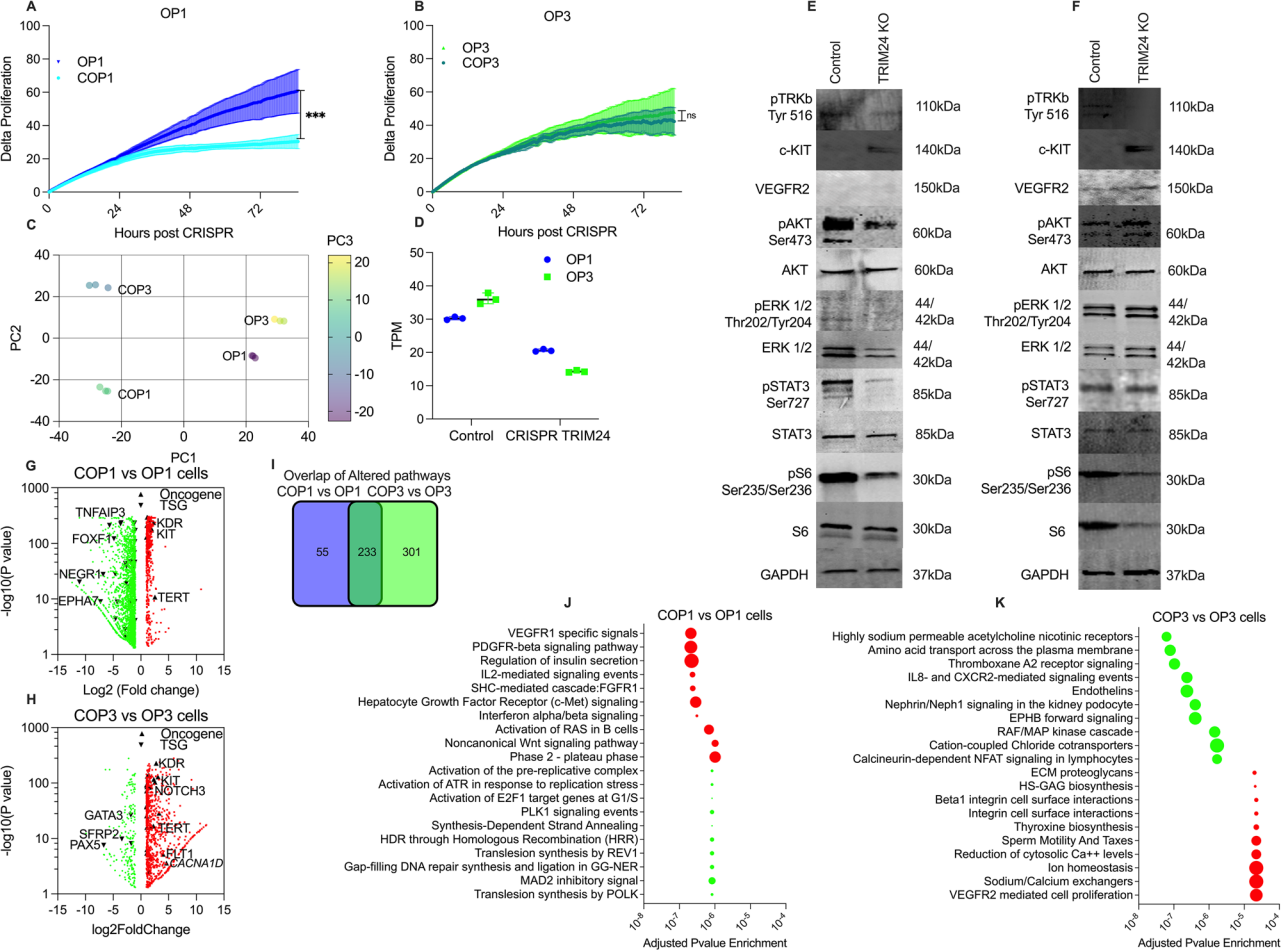
CRISPR Cas9 editing of primary cells resulted in diminished driver capacity of the fusion gene in OP3. The effect of *TRIM24::NTRK2* CRISPR editing on proliferation of OP1 (**A**) and OP3 (**B**). **C** The top 500 differentially expressed genes in COP3 vs OP3 and COP1 vs OP1 were clustered by principal component analysis. **D** The degree of CRISPR knockout was quantified by transcripts per million (TPM) of *TRIM24::NTRK2* transcript from RNA-seq expression data. The effects of CRISPR knockout on activation of TRKB TKD (pTRKB), downstream oncogenic signaling pathways (pERK1/2, pAKT, pSTAT3, and pS6), and c-KIT and VEGFR protein expression were quantified by western blot for OP1 (**E**) and OP3 (**F**). **G** Volcano plot showing differentially expressed genes (DEGs) to CRISPR knockout for COP1 vs OP1 with marked tumor suppressor genes (TSG) and oncogenes, and selected genes labeled and the specific pathways that were up- and downregulated in COP1. **H** Volcano plot showing DEGs for COP3 vs OP3 with marked TSG and oncogenes and selected genes labeled. **I** Pathway analysis shows a significant overlap in the altered pathways between COP1 vs OP1 and COP3 vs OP3. Bubble plot showing enrichment of specific genes up- and downregulated in COP1 vs OP1 (**J**) and COP3 vs OP3 (**K**). Downregulated (green) pathways, and upregulated (red) pathways with the number of nodes represented as bubble size.

To determine if the decreased efficacy of TRK inhibitor was due to drug resistance, the *TRIM24::NTRK2* fusion was knocked out (KO) by CRISPR-Cas9 editing in OP1 (COP1) and OP3 (COP3); adding three stop codons and a fluorescent tag into *TRIM24* exon 1. The inhibition of *TRIM24* in COP1 resulted in decreased proliferation versus the wt control OP1 (Fig. 4A), but no difference was observed in COP3 versus wt (Fig. 4B). Differential expression analysis by RNA-seq in the KO versus wt cell lines showed high consensus between the three experimental replicates and a clear relatedness of sample origin by PCA (Fig. 4C). Expression analysis also verified a successful knockout by significant decrease of *TRIM24::NTRK2* in COP1 and COP3 (Fig. 4D). Further, we determined the effects of CRISPR knockout of TRIM24::NTRK2 by quantifying the activation of TRKB and downstream oncogenic pathways. Active pTRKB was almost eliminated in both COP1 and COP3 cells. Considerable reductions in oncogenic signaling of pAKT, pSTAT3, and pS6 were observed in COP1, but there was no alteration in pERK (Fig. 4E/F). The knockout effect in COP3 was highly muted, with only S6 showing reduced activation (Fig. 4F).

Differentially expressed genes (DEGs) in COP1 versus OP1 were identified, and Volcano plots produced with known oncogenic and tumor suppressor genes (TSG) from the OncoKB cancer gene list marked. This revealed upregulation of known oncogenes; KDR, KIT, and TERT in the *TRIM24::NTRK2*-inhibited COP1 cells (Fig. 4G). These oncogenes were also upregulated in COP3 (vs OP3) along with *FLT1* and *NOTCH3,* showing a generalized increase in gene expression from COP1 to COP3 (Fig. 4H). The upregulation of c-KIT was verified on the protein level by Western Blot (Fig. 4E/F). There was a significant degree of concordance when investigating the altered signaling pathways in COP1 and COP3 by CTpathway enrichment from RNA expression data, showing 233 altered pathways overlapping between the two comparisons (COP1 vs OP1 and COP3 vs OP3). The COP3 (vs OP3) showed 301 unique signaling pathway alterations, whereas COP1 (vs OP1) revealed only 55 unique pathways (Fig. 4I). The top-altered pathways were identified for each of the COP vs OP groups. In COP1, the knockout of TRIM24::NTRK2 resulted in downregulated; DNA replication, repair mechanisms, and G1/S- phase transition. Meanwhile, the upregulated pathways included VEGFR1 specific signals, PDGFRβ signaling pathway, IL2 signaling, and non-canonical Wnt signaling (Fig. 4J). The pathways upregulated after TRIM24::NTRK2 knockout in COP3 were integrin signaling, ion homeostasis, and VEGFR2-mediated cell proliferation, and downregulated pathways included IL8 cytokine signaling and RAF-MAP signaling (Fig. 4K). The increased VEGFR2 expression in COP3 was verified by Western Blot (Fig. 4E/F). Taken together, these results indicated that the tumor became less reliant on the *TRIM24::NTRK2* oncogenic driver over time and had the capacity to activate other oncogenic signaling pathways.

### Expression analysis and pathway enrichment show oncogenic activity by other tumor drivers

**Fig. 5:**
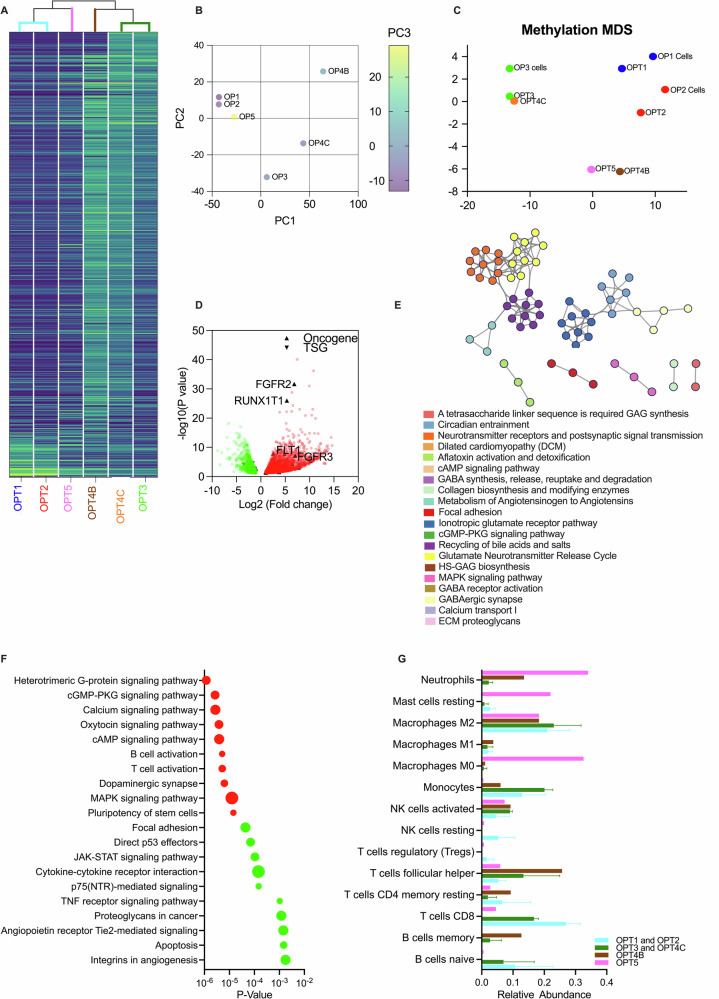
Expression analysis from RNA-sequencing data shows multiple oncogenic drivers becoming active over time in the tumor. **A** Heatmap of the top 5000 differentially expressed genes (DEGs) in six primary patient materials from five operations (OPT1-OPT5). **B** Principal components analysis (PCA) plot of the top 500 DEGs by RNA-seq in OPT1-OPT5. **C** PCA of the 1000 most differentially methylated regions for each of the operations and corresponding OP1-3 cell lines. **D** Volcano plots of DEGs between two tumor groups; OPT3/4C vs OPT1/2. Oncogenes and tumor suppressor genes (TSGs) have been highlighted, with *FGFR2* and *RUNX1T1* significantly upregulated in the OPT3/4C group. **E** Enrichment map of DEGs comparing two tumor groups; OPT3/4C and OPT1/2. **F** Pathway bubble plot showing the specific pathways that were up- (red) and downregulated (green) in OPT3/4 vs OPT1/2, with the number of nodes represented as bubble size. **G** Comparison of the estimated immune cell composition of OPT1/2, OPT3/4C, OPT4B, and OPT5 by CIBERSORTx on RNA-seq data.

To determine the differential expression over time a hierarchical clustering from the six patient samples was performed. The heatmap revealed two broad groupings including OPT1, OPT2 and OPT5 in one group and OPT3, OPT4C and OPT4B in a second group (Fig. 5A). Principal components analysis (PCA) revealed a tight clustering between OPT1 and OPT2 as well as OPT3 and OPT4C, whereas OPT5 clustered with OPT1/2 and OPT4B showed no clear affiliation (Fig. 5B). The methylation clustering also indicated dissimilarities in OPT4B and OPT5 to the other samples, and these two samples were therefore excluded from the differentially expressed gene (DEG) analysis (Fig. 5C). The two distinct subgroups, OPT1/2 (group 1) and OPT3/4C (group 2), were compared to each other. A volcano plot of the DEG matched to the OncoKB cancer gene list revealed upregulation of 44 known oncogenes and downregulation of 23 known TSGs in OPT3/4C vs OPT1/2. Among the upregulated oncogenes, several tyrosine kinases were identified, including *EGFR*, *FGFR1-3*, *FLT1*, *KIT*, *NTRK2*, and *MET* (Supplementary Table 5). The largest increase in expression was seen in the oncogenes *FGFR2* and *RUNX1T1* (Fig. 5D). Signaling pathway enrichment from DEGs by CTpathway revealed two distinct gene clusters: one included ionotropic glutamate receptor pathway and GABAergic synapse and the other incorporated neurotransmitter receptors and postsynaptic signal transmission (Fig. 5E). The downregulated pathways in OPT3/4C included direct p53 effectors, TNF-receptor signaling and apoptosis pathways mediators (Fig. 5F). The upregulated pathways in OPT3/4C included gene clusters involved in Heterotrimeric G-protein signaling, T-cell activation, B-cell activation, and MAPK signaling pathways (Fig. 5F).

To determine changes in the tumor microenvironment over time, the immune cell fractions were computed, distinguishing 22 different immune cells in bulk tumor tissue based on RNA-seq data. This showed that naïve B-cells and CD8 T-cells were enriched in OPT1/2 as well as in OPT3/4C but were diminished in OPT4B and OPT5 (Fig. 5G). M0 macrophages and neutrophils were increased over time. H&E slides were retrospectively examined (data not shown), analyzing blood vessel formation and the presence of immune cell infiltration. The OPT2, followed closely by OPT5, was the most vascular, indicating that the occipital lobe tumor was consistently more vascular in comparison to the parietal lobe tumor. Necrotic patches were revealed in OPT5, indicating rapid growth. In addition, clinical IHC analysis of OPT5 showed substantial CD8+ T-cell presence and moderate CD4+ T-helper cells in the perivascular area of the tumor, as well as CD3+ T-cells throughout the tumor (data not shown). To remove the effect of the immune component observed in the patient samples (OPT1 and OPT3) and further refine upregulated oncogenes, we compared the DEG data (OPT3/4C vs OPT1/2) to the cultured cell data (OP3 vs OP1). Using this methodology, eight highly expressed oncogenes were identified in group 2; *FOXF1*, *CACNA1D*, *CDH11*, *RUNX1T1*, *ONECUT2*, *FGFR2*, *FLT1*, and *MN1* (Supplementary Table 5). These results showed that the tumor gained additional oncogenic drivers and modified the immune cell composition throughout tumor progression.

## Discussion

We present a case of high-grade glioma in a 6-year-old patient with a rare *TRIM24::NTRK2*-fusion, having had five tumor relapses in 4 years. Methylation profiling indicated an PXA, but the tumor was eventually diagnosed as an epithelioid glioblastoma, WHO grade 4. Epithelioid glioblastoma was recognized as a new variant of glioblastoma in the 2016 WHO classification and falls under the umbrella of IDH wild-type glioma^3^. It does not have its own entity in the methylation classifier but is interspersed within PXA due to significant overlap. Among the variants found in the tumor, *CDKN2A/B* HD occurs in around 50% of epithelioid glioblastoma, and p*TERT* mutations are also common^20,21^. *CDKN2A* and *CDKN2B*, encoding p16 and p15, respectively, are TSGs with strong regulatory effects on the cell cycle and apoptosis. Homozygous deletion of these genes has been speculated to contribute to the emergence of off-target resistance to larotrectinib in TRK fusion-positive cases^22^. This is a common feature of *IDH* wild-type gliomas where it contributes to the aggressive nature of those tumors. There is no standard treatment to target *CDKN2A/B* deleted tumors, although current studies using CDK4/6 inhibitors are ongoing with a number of trials in HGG^23^, some showing limited success in specific HGG subgroups^24^. Due to the lack of established clinical data support and lack of available local clinical trials, CDK4/6 inhibitors were not considered for this patient.

NTRK alterations have been widely described in pediatric gliomas, including pHGG. One glioblastoma patient with a *NACC2::NTRK2* fusion was still in remission two years after treatment with conventional therapy^25,26^. Another patient with an Infant-type hemispheric glioma with a *ETV6::NTRK3* fusion was treated with larotrectinib and demonstrated response to treatment with tumor regression and clinical stability^27^. These case studies show promising management of TRK fusion positive gliomas with and without the use of targeted therapies in children. *TRIM24* has previously been identified as a rare 5’ fusion partner with *BRAF* and *MET* genes^18,28^ and has once been reported as a partner to *NTRK2* in one case with lung adenocarcinoma^29^ TRIM24 is a transcriptional regulator and potential oncogenic driver in glioma and has been shown to play a pivotal role in drug resistance, STAT3 mediated migration^30^, transcriptional control^31^, and maintenance of glioma stem cells^32^. However, our results point to *TRIM24::NTRK2* not being the only driver in this tumor, but is likely a combination with *CDKN2A/B* HD, p*TERT*, and other acquired alterations in later relapses. Interestingly, the only somatic nsSNV present in all tumor samples was a *SLC6A14* p.R614H variant of unknown significance (VUS). Although expression of this gene has been identified to correlate with migration in gastric cancer^33^, the R614H missense variant shows low pathogenicity scores, is not localized in any critical domain, and occurs at low frequency in the normal population, which makes it most likely a passenger mutation. Taken together, the alterations found in this case drive growth signaling, cell cycle, transcriptional programming, and immortalization, which, when combined, likely have a collaborative effect maintaining tumor growth.

In this patient, we identified two genetically distinct tumor clones. Due to the divergent OPT3 and OPT4C specimens, exhibiting slower growth and abundant DNA double-stranded breaks and numerous of SNVs and SVs compared to the sustained genomic profiles of OPT2, OPT4B, and OPT5, we propose that the initial radiation therapy led to divergent tumor lineage formation. When the MRI was combined with the irradiation field from the initial treatment (Fig. 1B), showing the OPT2 relapse, approximately half of the relapsed tumor arose within the region where full radiation dose was administered. This supports the theory that the first relapse (OPT2) grew from outside of the full-dose irradiated area, while OPT3 and OPT4C grew from within this zone.

We speculate that the new acquired radiation-derived alterations in OPT3 and OPT4C replaced the TRK fusion as the primary oncogenic drivers, while OPT2, OPT4B, and OPT5 remained dependent on the TRIM24::NTRK2 fusion and eventually acquiring TRKi resistance alterations. The MTOR p.L1433S mutation, found in OPT4B, is known to cause resistance after third-generation EGFR inhibitor treatment in non-small cell lung cancer (NSCLC)^34^, and functional analysis of MTOR p.L1433S is found to induce mTORC1 pathway activity^35^. The NTRK2 p.G639R variant, found in OPT5, is a “solvent front” mutation that prevents inhibitor binding and causes resistance to first-generation TRK-inhibitors (larotrectinib and entrectinib)^36^. Yet, the second-generation TRK-inhibitors (repotrectinib) have shown efficacy against tumor cells with NTRK2 p.G639R substitution in preclinical studies^37^, but unfortunately did not have clinically relevant effect in this patient. These resistance issues may be avoided with next-generation TRK inhibitors like zurletrectinib, which have shown early in vitro promise in cell models^38^.

A general increase in resistance to larotrectinib and other TRK inhibitors could be observed in the patient-derived cells over time, which is a common trait of glioma stem cells^37^. This may partly be due to the heterogenic nature of the glioma stem cells, but also an elevation of drug transporters, such as ABCB1, that has been shown to be involved in acquired general drug resistance in cancer cells^36,39,40^. In addition, the lack of efficacy targeting the fusion gene in OPT3, OPT4, and OPT5 may result from increased activation of the AKT-MAPK pathways modulated by additional membrane-bound kinase receptors. We observed strong phosphorylation in the RAS and MAPK signaling in the OPT3, OPT4C, and OPT4B material despite TRK, MEK, and mTOR inhibition in the patient. Additionally, in vitro analyses revealed that although the fusion gene was inhibited (CRISPR or TRKi), there was preservation of AKT phosphorylation. We identified two membrane-bound receptors that could account for this para-activation: *FGFR2* and *FLT1*. The expression of these genes was increased in the later tumors, suggesting that despite clonal differences, the tumor converges on similar signaling pathways over time. *FLT1* and *FGFR2* encode receptors of the vascular endothelial growth factor (VEGFR1) and fibroblast growth factor (FGFR2), and both have approved precision treatments (FGFR2/erdafitinib and VEGFR1/bevacizumab). CRISPR knockout of the *TRIM24::NTRK2* fusion gene also showed tumor reliance on the VEGFR pathway. Given the rapid progression after OPT5, the relatively low FGFR2 expression detected in OPT5, and strong pathway activation of VEGFR, bevacizumab was combined with larotrectinib and sirolimus as a last treatment effort.

In conclusion, this case demonstrates the challenges in selecting appropriate treatments for pediatric high-grade gliomas due to progression and subclonal evolution. It is essential to initiate precision treatments directly as suitable targets are identified, in conjunction with standard therapy. However, cell heterogeneity, acquisition of additional oncogenic alterations reduce the successful outcome of targeting single alterations. Therefore, combinatorial treatments compensating also for loss of function mutations (e.g. CDK4/6 inhibition for homozygous *CDKN2A/B* deletion) and targeted elimination of glioma stem cells before clonal evolution occur, could increase treatment success in these tumor entities. These nuanced approaches will hopefully assist in preventing resistance adaptations to therapies and allow for a greater understanding of the interplay between genetic alterations, tumor heterogeneity, treatment resistance, and the tumor microenvironment.

## Material

### Patient material

Fresh frozen tumor material from the patient was obtained from each operation (OPT1-OPT5). Patient-derived cell lines were established from fresh tumor tissue collected in PBS from operations 1, 2, and 3 (referred to as OP1, OP2, and OP3 cells). During the fourth operation (OP4/OPT4), two tumor biopsies were obtained, herein termed basal (B) and cranial (C). The patient’s parents provided informed consent for participating in this study, and the Medical Ethics Committee of the Sahlgrenska University Hospital, Gothenburg (No. 239-13 to F Abel) the regional ethics committee in Västra Götaland (No. 604-12 to H Carén), Sweden, approved the study.

### Clinical routine analysis

Hematoxylin and eosin (H&E) staining of formalin-fixed paraffin-embedded (FFPE) tumor sections was performed for routine pathological examination and assessment of tumor cell morphology in OPT1-OPT5. The primary tumor tissue (OPT1) was assessed by immunohistochemistry (IHC) for *MAP2, SOX10, GFAP, TP53, INI1*, as well as mutated *IDH1* and *BRAF*. Mutations in *BRAF, KRAS*, *NRAS*, and *EGFR* were assessed by Ion AmpliSeq Colon and Lung Cancer Research Panel v2 with IonS5XL sequencing (Thermo Fisher Scientific, Waltham, MA, USA). Tumor material (FFPE) was sent for external evaluation to Bonn, Germany, and subsequently analyzed for fusion genes by a NanoString panel including 88 fusion transcripts. Immune cell content was assessed by IHC in OPT5 by routine analysis for CD3, CD4, CD8, and CD20.

### DNA methylation analyses

DNA (500 ng) extracted from FFPE tumor tissue or established cell lines (OP1-3) was bisulfite converted with EZ DNA methylation kit (Zymo Research, Irvine, CA, USA) as described earlier^41^. Genome-wide DNA methylation profiles were generated with the Infinium Methylation EPIC Bead-Chip array v1 or v2 (Illumina, San Diego, CA, USA) and analyzed as previously described^42^. The classifications were made using the MNP Classifier version 12.5 or 12.8 (www.molecularneuropathology.org). Multidimensional scaling analysis was processed and normalized with Noob normalization using R and the packages ChAMP1 and Minfi^43^.

### Whole genome sequencing and RNA sequencing

Paired whole genome sequencing (WGS) by Illumina TruSeq DNA PCR-Free kit was performed using 1 µg DNA from fresh frozen tumor tissue or cultured cells (90x coverage) and the patients normal blood lymphocytes (germline, 30x coverage) as part of the Genome Medicine Sweden (GMS) project^10^. Somatic variant calling was performed as follows; single nucleotide variants (SNVs) and small insertions/deletions (indels) by TNscope (Sentieon), structural variants (SVs) by Manta, and copy-number variant (CNV) changes by Canvas. Variants were filtered and classified in QCI Interpret (Qiagen), and checked in Integrative genomics viewer (IGV) as previously described in ref. ^10^. Whole mRNA sequencing (mRNA-seq) was performed on 250–500 ng RNA extracted from fresh frozen tumor tissue (single experiments) or cultured cells (three experimental replicates) by Illumina Stranded mRNA Prep, as previously described in ref. ^10^. Reads were mapped to the GRCh38 genome by STAR, gene fusion detection by Arriba (v2.1.0). Only in-frame coding fusion transcripts of clinical significance were kept. Transcript-level abundance from RNA-seq read data was calculated by Salmon and normalized to Transcripts Per Million (TPM). Immune cell fractions were computed using CIBERSORTx and the LM22 signature matrix^44^. Differential gene expression was analyzed with DESeq2, considering genes with a *p* value < 0.05 and a log2 fold > 1 as significantly deregulated. Differentially expressed genes were compared to the OncoKB list of 1172 known oncogenes and tumor suppressor genes (TSGs) (https://www.oncokb.org/cancer-genes, 19/2/2024; RRID:SCR_014782). Pathway enrichment analysis was performed with CTpathway^45^. PCA of differentially expressed genes was performed with plotPCA in DESeq2 (RRID:SCR_000154).

### FISH and Archer fusionPlex

FFPE tumor sections were assessed for NTRK2 rearrangement by Interphase FISH and NTRK2 break-apart probe (ZytoLight SPEC NTRK2 Dual Color Break Apart Probe, ZytoVision GmbH, Bremerhaven, Germany), and targeted open-end RNA sequencing by Archer FusionPlex Pan Solid Tumor v2 panel (targeting 137 genes; eu.idtdna.com) as previously described^46^.

### Immunohistochemistry

Tumor sections (4 μm) from OPT1-OPT5 were assessed by Immunohistochemistry (IHC) by antibodies targeting proteins in the MAPK-, PI3K-, and JAK/STAT- pathways (Supplementary Table 1) as previously described in ref. ^47^. Non-neoplastic FFPE brain tissue and tumor tissue with omitted primary antibodies were used as control.

### Cell Culture

Cell cultures were established from fresh tumor biopsies from 3 operations (OP1-3) by dissociation into single cells using a previously established protocol^48^. The patient-derived cell lines tested negative for mycoplasma by qPCR performed externally (Eurofins Genomics, Ebersberg, Germany). Human embryonic kidney cells (HEK293; RRID:CVCL_0045) were obtained from the ATCC Cell Line Collection (Manassas, VA, USA). HEK293 was maintained in DMEM supplemented with 10% FBS, 1% L-Glutamine, 1% HEPES solution, and 1% sodium pyruvate.

### Cell transfections

Three different constructs were generated using the pCMV6-Myc-DDK vector; pCMV6-NTRK2-Myc-DDK (NTRK2^WT^), pCMV6-TRIM24-NTRK2-Myc-DDK (*TRIM24::NTRK2* fusion), and pCMV6-Myc-DDK (empty vector). The wild-type NTRK2 (NM_006180, 838 aa, #RC221794) and pCMV6 empty vector (#PS10000) constructs were ordered from Origene (Origene, Rockville, MD, USA). Vector constructs for the *TRIM24::NTRK2* fusion transcripts exon 12–13 (1044 aa), and exon 12-14 fusion (1028 aa) were synthesized, subcloned, and sequenced by Invitrogen GeneArt (Thermo Fisher Scientific) HEK293 cells were transfected as previously described^47^.

### Western blot

Protein lysates (50 µg/sample) were loaded onto Mini-PROTEAN® TGX™ any KD (BioRad Laboratories, Hercules, CA, USA) according to the manufacturers protocol, as previously described^47^. The primary antibodies (Supplementary Table 1) were diluted in PBST (0.1% Tween-20 in PBS). Fluorescent band intensities in each sample were normalized against the intensity of total loaded protein from stain-free gel images. GAPDH was included to visualize loading. The ratio of normalized band intensities for phosphorylated proteins, pERK, pAKT, pSTAT3, pS6, and pTRKB, relative to the normalized band intensities of total ERK, total AKT, total STAT3, total S6, and DDK tag (DYK) protein quantities, respectively, was calculated for each sample. Fold changes of the ratios were calculated relative to the mean of NTRK2^WT^.

### Dose-response curves, drug synergy evaluation and cell viability assays

Evaluation of targeted treatments and identification of the best combination was established from three tropomyosin receptor kinase inhibitors (TRKi) targeting TRKA/B/C fusion proteins, in combination with trametinib (MEK1/2 inhibitor) and everolimus (mTOR inhibitor) were compared; first generation TRKi:s larotrectinib and entrectinib, second generation TRKi repotrectinib (Supplementary Table 2). All inhibitors were purchased from Selleck Chemicals GmbH (Germany).

Dose-response curves were optimized for each inhibitor and the three cell lines. Each inhibitor was prepared as 1 mM stock diluted in 100% DMSO and diluted to a span of ten concentrations, with 10 µM being the highest (DMSO conc 0.1%) in medium. Plates were incubated for 96 h and monitored by the live-imaging system Incucyte (Essen Bioscience, UK). Viability was determined using a luminescence-based, CellTiter-Glo 2.0 cell viability assay, using a Glomax Discoverer (Promega, Madison, USA).

The synergy screen was based on the IC50 of OP1 cells and OP3 cells. From these, a high (~IC50 of OP3) and a low (~IC50 of OP1) dose of each inhibitor was selected (Supplementary Table 3). Following the same seeding protocol, the plates were incubated for 96 h, monitored by the Incucyte, and viability was determined with Cell-Titer Glo. Protein was extracted from 12-well plates seeded at 60,000 cells per well after 96 h of drug treatment. The cells were harvested, pelleted, and protein was extracted by aspirating the media and incubating on ice for 5 min, followed by adding ice-cold RIPA buffer.

### CRISPR Cas9 editing of TRIM24::NTRK2

Patient-derived OP1 and OP3 cells were modified by adding a fluorescent tag and 3 stop codons using TrueGuide^TM^ Synthetic sgRNA for TRIM24 exon 1 (ACACGGCGCAAGTGTCCAAC) or TrueGuide^TM^ Synthetic sgRNA Negative Control (AAAUGUGAGAUCAGAGUAAU) with no predicted off-target cuts, by lipofectamine Crisprmax CAS9 transfection reagent, according to the manufacturer’s instructions (Thermo Fisher Scientific). Briefly, wells were seeded in a 6-well plate at 1.5 × 10^5^ cells/well, grown to a confluency of 70% and then genetically modified. The cells were fluorescently visualized 48 h post-editing to confirm the editing success. The editing experiment was performed as three separate experimental replicates.

### Proliferation

Cell proliferation of HEK293, OP1 wild-type (wt) and OP1 CRISPR-modified cells (COP1), and OP3 wt and OP3 CRISPR-modified cells (COP3) was measured with an RTCA real-time cell monitoring platform (Agilent) using E16 plates^49^. Briefly, 1 × 10^4^ cells/well were seeded and allowed to grow for 24 h. Once established, the cells were subsequently transfected using the previously described methodology. Impedance was measured at 1-min intervals over 72 h and normalized against the impedance at the start of the transfection experiment to give the impedance change over time (Delta Proliferation).

### Statistical analysis

Proliferation changes were calculated by the total area under the curve (AUC) per experiment with four technical replicates and presented as the mean and SEM of three independent experiments over time. Western blots were analyzed by ordinary one-way ANOVA test followed by Dunnett´s multiple comparison test. Analysis of drug treatments was conducted in R, and figures were produced using the package ggplot2. Drug combination effects were estimated using the Bliss interaction score^50^, and the significance level of the interaction was estimated with ANOVA. The dose-response curves were fitted with a 3-parameter log-logistic model, and the IC50 was estimated using the R package drc^51^. Welch’s two-sided *t*-test was used to test for differences in IC50 between the cell lines. Calculated significance; *p < 0.05, **p < 0.01, ***p < 0.001. All analyses use GraphPad Prism version 10.2.2 for MacOS (GraphPad Software, www.graphpad.com; RRID:SCR_002798).

## Supplementary information

Supplementary information

## Data Availability

Data is available on request.

## Code availability

No code was created for this study.
