# Supplementary material for: Comprehensive characterization and targeted treatment of a pediatric epithelioid glioblastoma with a rare TRIM24-NTRK2 fusion
Source: NPJ Precis Oncol. 2025 Dec 2;9:394. doi: 10.1038/s41698-025-01190-3 (PMC12678440; doi:10.1038/s41698-025-01190-3)
Supplement: Supplementary file 1 — Supplementary information [file 41698_2025_1190_MOESM1_ESM.docx]

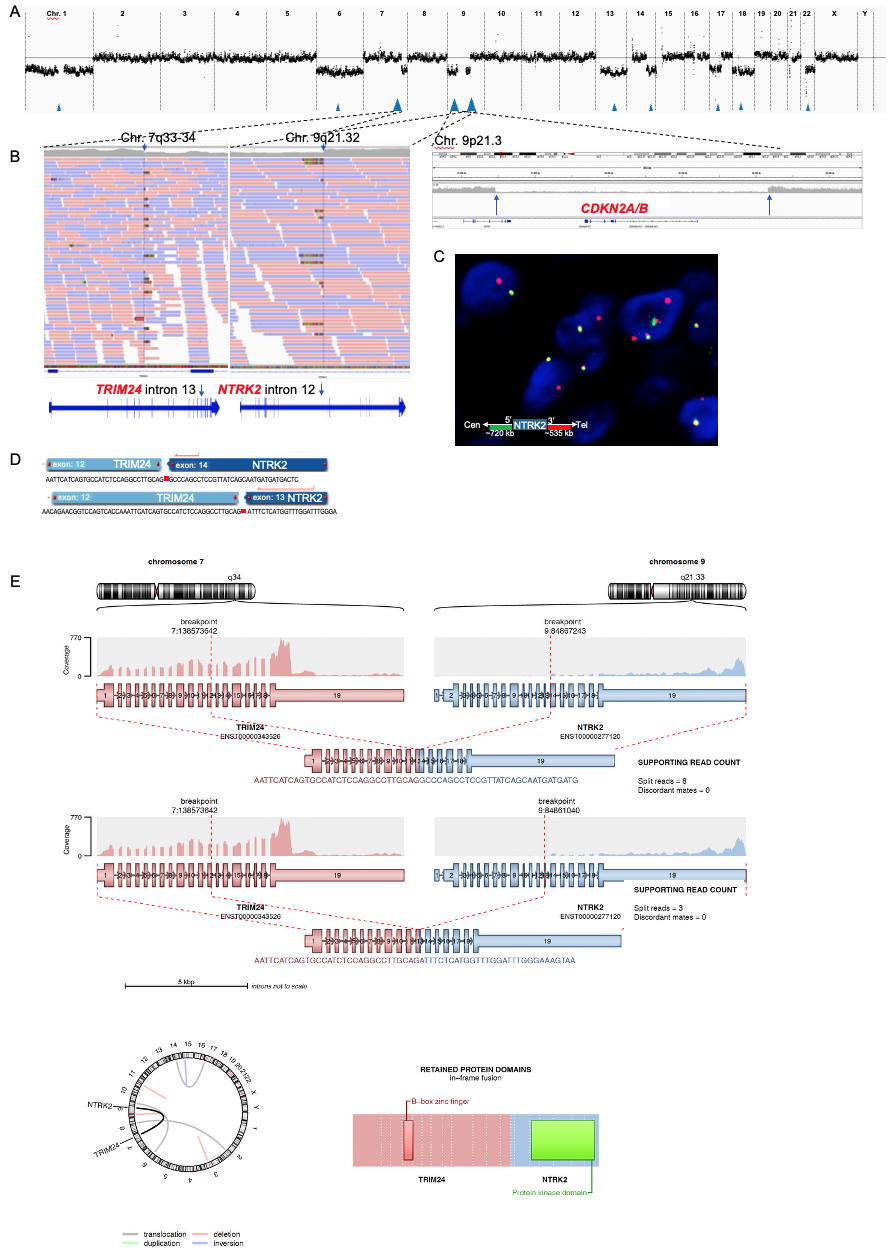


**Supplementary Figure 1.** **Identification and location of TRIM24::NTRK2 fusion gene.**
(A) Genome copy-number profile of the primary tumor from the patient. (B) Structural variant screening with Manta revealed a translocation between 7q33-34 and 9q21.32 with break points within *TRIM24* intron 13 (chr7:138577080) and *NTRK2* intron 12 (chr9:84847247), leading to a *TRIM24::NTRK2* fusion gene. (C) The rearrangement was verified in FFPE tumors sections by FISH on interphase cells using break apart probes for the *NTRK2* gene; 5´green probe and 3´red probe. Tumor cells show one normal wild type (wt) copy of *NTRK2* (merged orange signal) and one lone 3´ *NTRK2* part (red) corresponding to the fusion rearrangement (5´ *NTRK2* part is deleted). (D) The *TRIM24::NTRK2* fusion gene was verified in FFPE tumor tissue by Archer FusionPlex RNA-panel, showing two in-frame transcripts; exon 12-14 and exon 12-13 (3087nt and 3135nt long; NM_015905.3 and NM_006180.6, respectively). (E) The two in-frame transcripts of *TRIM24::NTRK2* were also found in fresh frozen tissue by whole mRNA sequencing using the Arriba fusion detection tool (fusion points: chr7:138573642-chr9:84867243, chr7:138573642-chr9:84861040) leading to fusion proteins of 1028 and 1044 amino acids with retained tyrosine kinase domain. Ensembl-ID ENST00000343526 correspond to NM_015905.3 and ENST00000277120 correspond to NM_006180.6 for TRIM24 and NTRK2 transcripts. The wt proteins of TRIM24 (NP_056989.2) NTRK2 (NP_006171.2) are 1050 and 838 amino acids.


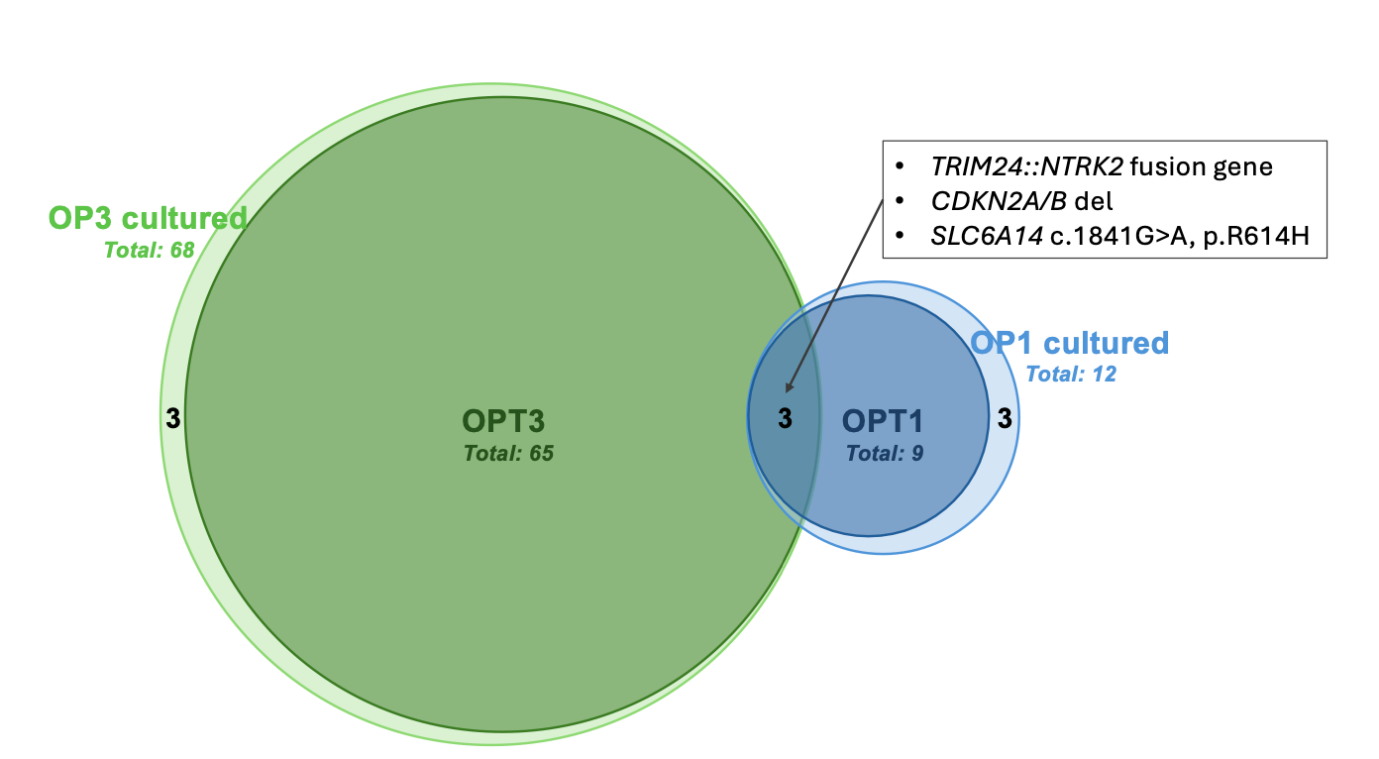


**Supplementary Figure 2. The genetic concordance of primary tissue and cultured cell lines.**

The comparison of nonsynonymous SNV/indels and all structural variants in tissue from operation 1 (OPT1), dark blue, cultured operation 1 cells (OP1, light blue), operation 3 (OPT3), dark green and cultured operation 3 cells (OP3, light green) showing the congruency between the tissue harvested from the same operation and the three common alterations shared between the two.


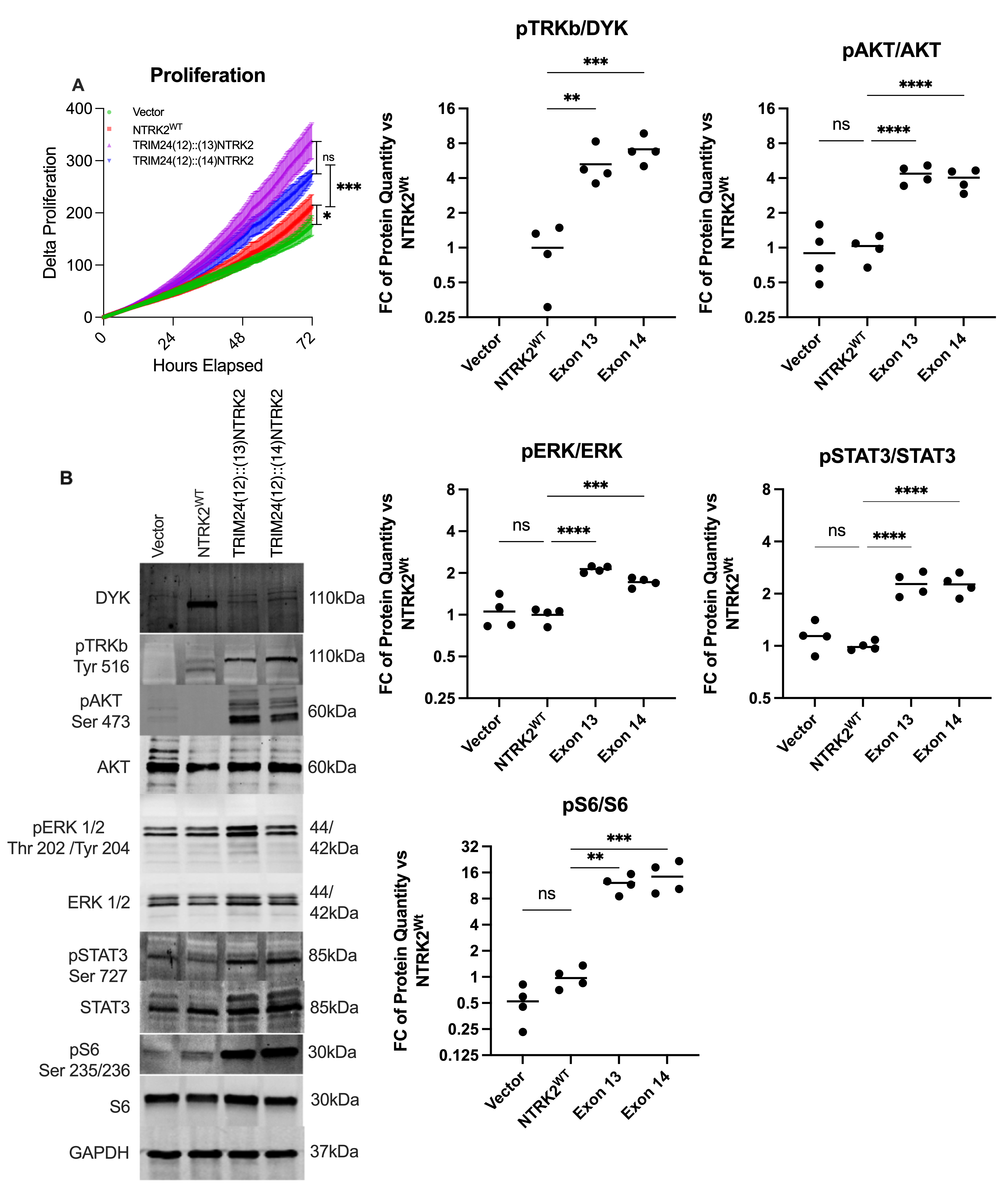


**Supplementary Figure 3. *TRIM24::NTRK2* functions as an oncogenic driver in HEK293. (**A) Proliferation was measured over 72 hours with the *TRIM24::NTRK2* fusion constructs on pCMV6 Myc-DYK backbone, resulting in increased proliferation compared to *NTRK2*^WT^ on pCMV6 Myc-DYK backbone. (B) The protein expression analysis of the two *TRIM24::NTRK2* constructs resulted in increased TRKB, AKT, ERK, STAT3, and S6 signaling. The quantification of phosphorylated TRKB tyrosine kinase activity, pAKT (PI3K pathway), pERK1/2 (MAPK pathway), pSTAT3 (JAK-STAT pathway) and ribosomal protein pS6 (MAPK and PI3K) showed a considerable upregulated activity of all three markers in both *TRIM24::NTRK2* constructs compared to *NTRK2*^WT^.

**
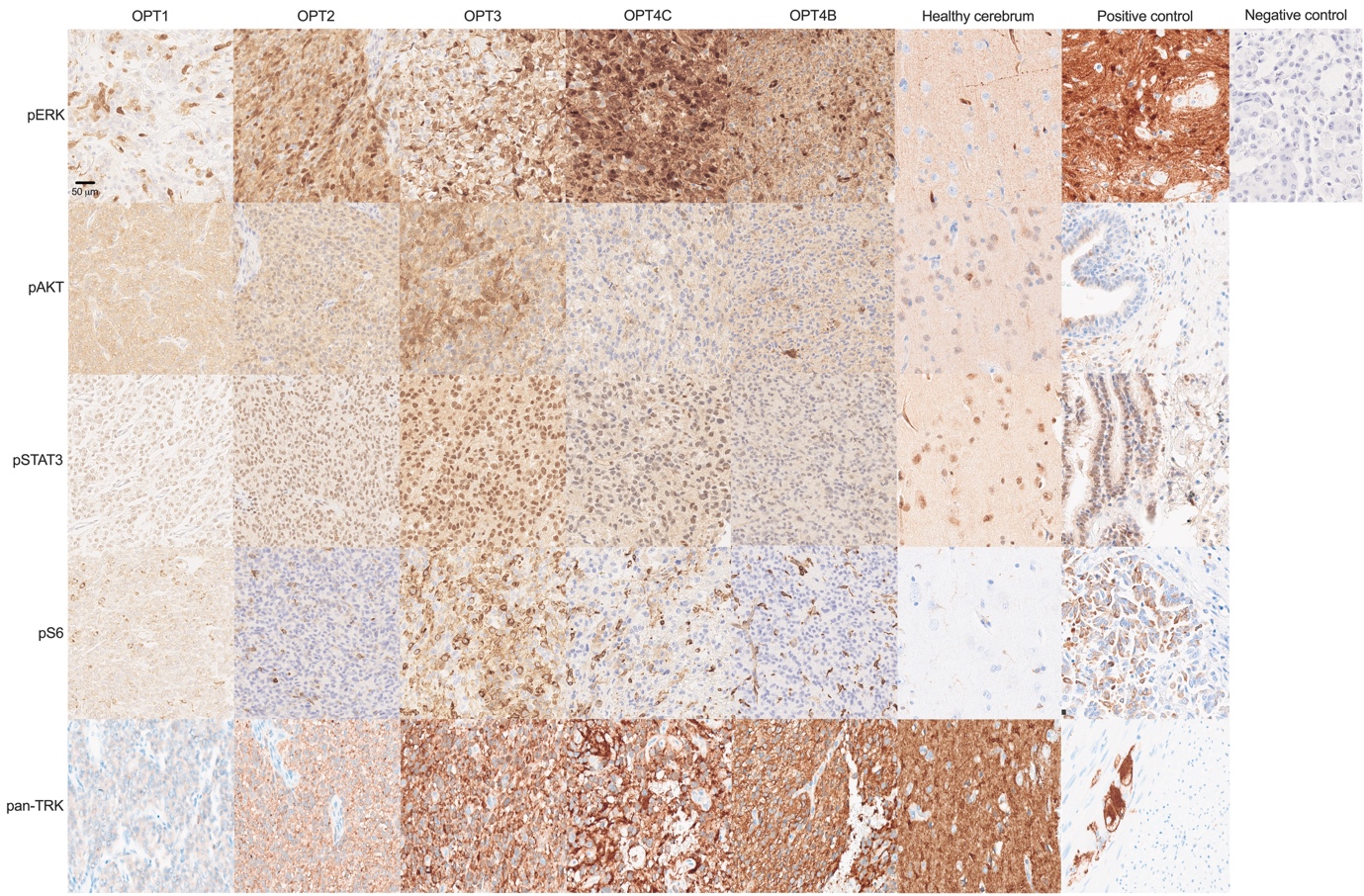
**

**Supplementary Figure 4. Immunohistochemical staining of MAPK, PI3K, and STAT3 signaling in five materials taken from four operations.** IHC for pERK, pAKT, pSTAT3, pS6 and pan-TRK confirmed the activation of the MAPK, PI3K, and STAT3 pathways in FFPE tumor sections from the first four operations (OPT1, OPT2, OPT3, OPT4C and OPT4B) compared to healthy cerebrum and positive controls; BRAF fusion positive Pilocytic astrocytoma (pERK), normal urothelium (pAKT), ROS1 fusion positive Lung adenocarcinoma (pSTAT3), normal urothelium (pS6) and ganglion cells in appendix (pan-TRK).

**Supplementary Table 1. Primary antibodies and dilutions used for IHC and Western blot experiments.**

| **Antibody** | **Manufacturer** | **Product number** | **Western Blot dilution** | **IHC dilution** |
| --- | --- | --- | --- | --- |
| VEGF Receptor 2 Monoclonal Antibody (B.309.4) | Invitrogen | MA5-15157 | 1:1000 |  |
| c-Kit Recombinant Rabbit Monoclonal Antibody (HC34LC14) | Invitrogen | 701494 | 1:1000 |  |
| Phospho-Erk1/2, Thr202/Tyr204, D13.14.4E XP® | Cell Signaling Technology | 4370 | 1:1000 | 1:2000 |
| p44/42 MAPK (Erk1/2) | Cell Signaling Technology | 4695 | 1:1000 |  |
| Phosphorylated-AKT, Ser473 | Cell Signaling Technology | 9271S | 1:1000 | 1:50 |
| Total Akt-1 | Cell Signaling Technology | 9272S | 1:1000 |  |
| phosphorylated-STAT3 Ser727 | Invitrogen | 44-384G | 1:1000 | 1:500 |
| total STAT3 | Invitrogen | MA1-13042 | 1:1000 |  |
| Phospho-S6 Ribosomal Protein (Ser235/236) (D57.2.2E) XP® | Cell Signaling Technology | 4858 | 1:1000 | 1:1000 |
| S6 Ribosomal Protein | Cell Signaling Technology | 2317 | 1:1000 |  |
| DYKDDDK tag (FLAG) | Invitrogen | MA1-142 | 1:1000 |  |
| Phospho-TrkB (Tyr516) | Invitrogen | PA5-38077 | 1:1000 |  |
| hFAB Rhodamine anti-GAPDH | BioRad | 12004168 | 1:2500 |  |

**Supplementary Table 2: Selected drugs, targets, and dose ranges for dose-response curves.**

| **Drug** | **Target** | **Dose Range** |
| --- | --- | --- |
| larotrectinib (#S5860) | ATP competitive, highly selective TRK-inhibitor | 10 µM à 0.1 nM |
| entrectinib (#S7998) | Pan-TRK A/B/C, ROS1 & ALK inhibitor | 10 µM à 0.1 nM |
| repotrectinib (#S8583) | ALK/ROS1/TRK inhibitor, also inhibits SRC | 10 µM à 0.01 nM |
| everolimus (#S1120) | mTOR inhibitor of FKBP12 | 10 µM à 1 nM |
| trametinib (#S2673) | Highly specific and potent MEK1/2 inhibitor | 10 µM à 0.1 nM |

**Supplementary Table 3: Selected drug doses for synergy screen**

| **Drug** | **High Dose (µM)** | **Low Dose (µM)** |
| --- | --- | --- |
| larotrectinib (#S5860) | 0.1 | 0.01 |
| entrectinib (#S7998) | 0.01 | 0.0001 |
| repotrectinib (#S8583) | 0.01 | 0.0001 |
| everolimus (#S1120) | 10 | 2.5 |
| trametinib (#S2673) | 0.05 | 0.01 |

**Supplementary Table 4: Result from Bliss Independence Model on Synergy Screen**

| **High Dose. Op1_c** | **p.val** | **interaction. score** | **Antagonistic (A)/ Synergistic (S)** | **Low Dose. Op1_c** | **p.val** | **interaction. score** | **Antagonistic (A)/ Synergistic (S)** |
| --- | --- | --- | --- | --- | --- | --- | --- |
| larotrectinib+everolimus | 1.40E-06 | 0.129 | A | larotrectinib+everolimus | 1.78E-05 | 0.161 | A |
| larotrectinib+trametinib | 3.10E-06 | 0.154 | A | larotrectinib+trametinib | 2.74E-03 | 0.219 | A |
| trametinib+everolimus | 5.14E-03 | 0.11 | A | trametinib+everolimus | 3.01E-02 | 0.174 | A |
| entrectinib+everolimus | 2.00E-07 | 0.185 | A | entrectinib+everolimus | 1.00E-07 | 0.187 | A |
| entrectinib+trametinib | 4.90E-06 | 0.204 | A | entrectinib+trametinib | 6.50E-04 | 0.261 | A |
| repotrectinib+everolimus | 1.50E-06 | 0.132 | A | repotrectinib+everolimus | 1.14E-02 | 0.138 | A |
| repotrectinib+trametinib | 2.70E-06 | 0.169 | A | repotrectinib+trametinib | 4.36E-03 | 0.272 | A |
| **High Dose. Op3_c** | **p.val** | **interaction. score** | **Antagonistic( A)/ Synergistic (S)** | **Low Dose. Op3_c** | **p.val** | **interaction. score** | **Antagonistic( A)/ Synergistic (S)** |
| larotrectinib+everolimus | 3.90E-06 | 0.146 | A | larotrectinib+everolimus | 1.09E-05 | 0.095 | A |
| larotrectinib+trametinib | 9.87E-05 | 0.165 | A | larotrectinib+trametinib | 6.97E-02 | 0.116 | A |
| trametinib+everolimus | 3.75E-04 | 0.146 | A | trametinib+everolimus | 4.57E-02 | 0.122 | A |
| entrectinib+everolimus | 3.59E-05 | 0.131 | A | entrectinib+everolimus | 1.12E-06 | 0.186 | A |
| entrectinib+trametinib | 4.19E-04 | 0.141 | A | entrectinib+trametinib | 1.11E-03 | 0.275 | A |
| repotrectinib+everolimus | 2.27E-05 | 0.132 | A | repotrectinib+everolimus | 8.60E-07 | 0.143 | A |
| repotrectinib+trametinib | 8.29E-05 | 0.179 | A | repotrectinib+trametinib | 3.84E-03 | 0.219 | A |

**Supplementary Table 5: Regulated OncoKB Genes in the OPT3/OPT4C tissue and cultured cells.**

| Oncogenes upregulated in OPT3/4C (vs OPT1/2) | TSGs downregulated in OPT3/4C vs OPT1/2 | Oncogenes upregulated in OP3 cells (vs OP1) | TSGs downregulated in OP3 cells (vs OP1) | Overlap of upregulated Oncogenes between OPT3/4C and OP3 cells |  |
| --- | --- | --- | --- | --- | --- |
| BCL2 | SOCS1 | CARD11 | GATA3 | FGFR2 | |
| BRAF | TNFAIP3 | FGFR2 | PRDM1 | FLT1 | |
| EGFR | B2M | CCND2 | GRIN2A | RUNX1T1 | |
| FGFR2 | FAS | HGF | CIITA | MN1 | |
| FGFR3 | CDKN1A | NTRK1 | CBFA2T3 | CACNA1D | |
| JAK1 | HLA-A | EPHB1 | PTPRD | CDH11 | |
| KIT | SDHAF2 | FGF19 | TRAF5 | FOXF1 | |
| MET | SH2B3 | FLT1 | SLFN11 | ONECUT2 | |
| MPL | TMEM127 | IL7R |  |  | |
| PAX5 | CD58 | INHBA |  |  | |
| WT1 | DUSP4 | NOTCH3 |  |  | |
| ERBB4 | FHIT | RUNX1T1 |  |  | |
| ERG | HLA-B | CD70 |  |  | |
| NTRK2 | IRF1 | COL1A1 |  |  | |
| AKT3 | NTHL1 | IGF2 |  |  | |
| FLT1 | PMAIP1 | MN1 |  |  | |
| IGF1R | SHQ1 | RAC2 |  |  | |
| REL | IRF2 | ACKR3 |  |  | |
| SETBP1 | MAD2L2 | CACNA1D |  |  | |
| TERT | ROBO1 | CDH11 |  |  | |
| TP63 | SOCS3 | FOXF1 |  |  | |
| EPHA7 | DDX41 | GATA6 |  |  | |
| RRAS2 | KLF2 | MERTK |  |  | |
| RUNX1T1 |  | ADHFE1 |  |  | |
| CYSLTR2 |  | FGF1 |  |  | |
| FLI1 |  | FOLH1 |  |  | |
| MN1 |  | ONECUT2 |  |  | |
| PAX3 |  |  |  |  | |
| PGR |  |  |  |  | |
| APLNR |  |  |  |  | |
| ARHGEF28 |  |  |  |  | |
| CACNA1D |  |  |  |  | |
| CDH11 |  |  |  |  | |
| ETS1 |  |  |  |  | |
| FOXF1 |  |  |  |  | |
| KSR2 |  |  |  |  | |
| MGAM |  |  |  |  | |
| PIK3C2B |  |  |  |  | |
| STAT4 |  |  |  |  | |
| TAF1 |  |  |  |  | |
| LGR5 |  |  |  |  | |
| ONECUT2 |  |  |  |  | |
| POU3F4 |  |  |  |  | |
| REV3L |  |  |  |  | |

Western blot raw images

CRISPR OP1


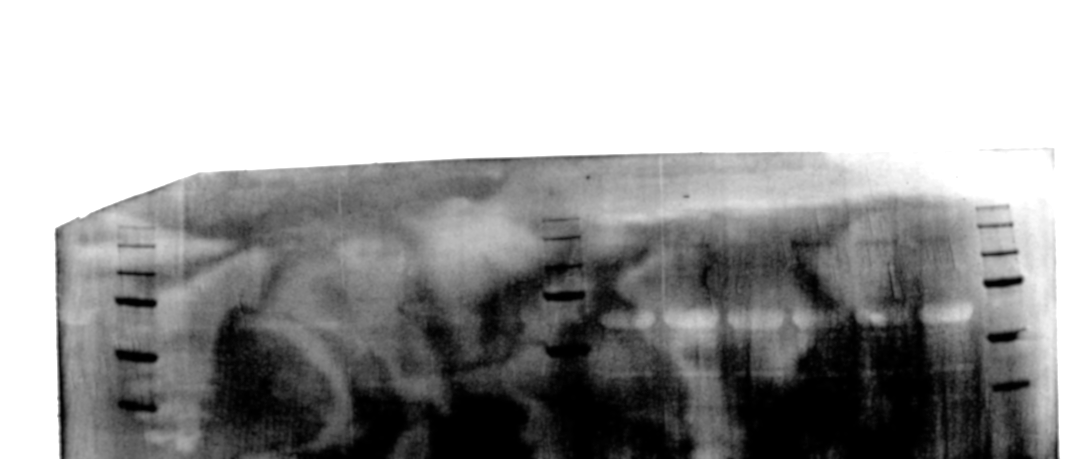


110Kda

pTRKb


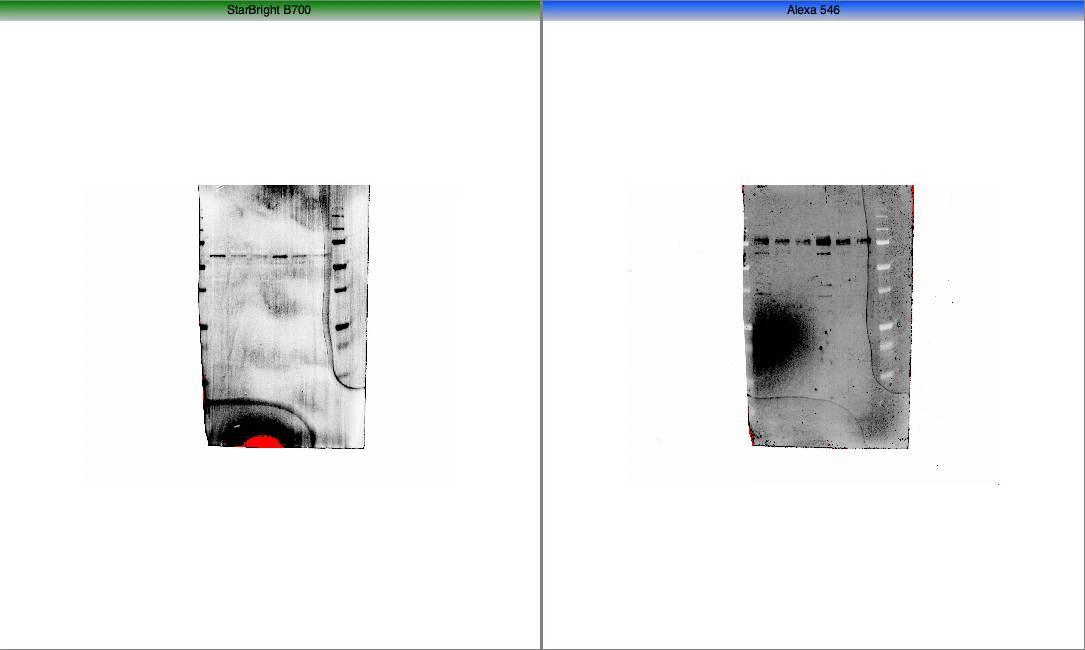


60Kda
pAKT


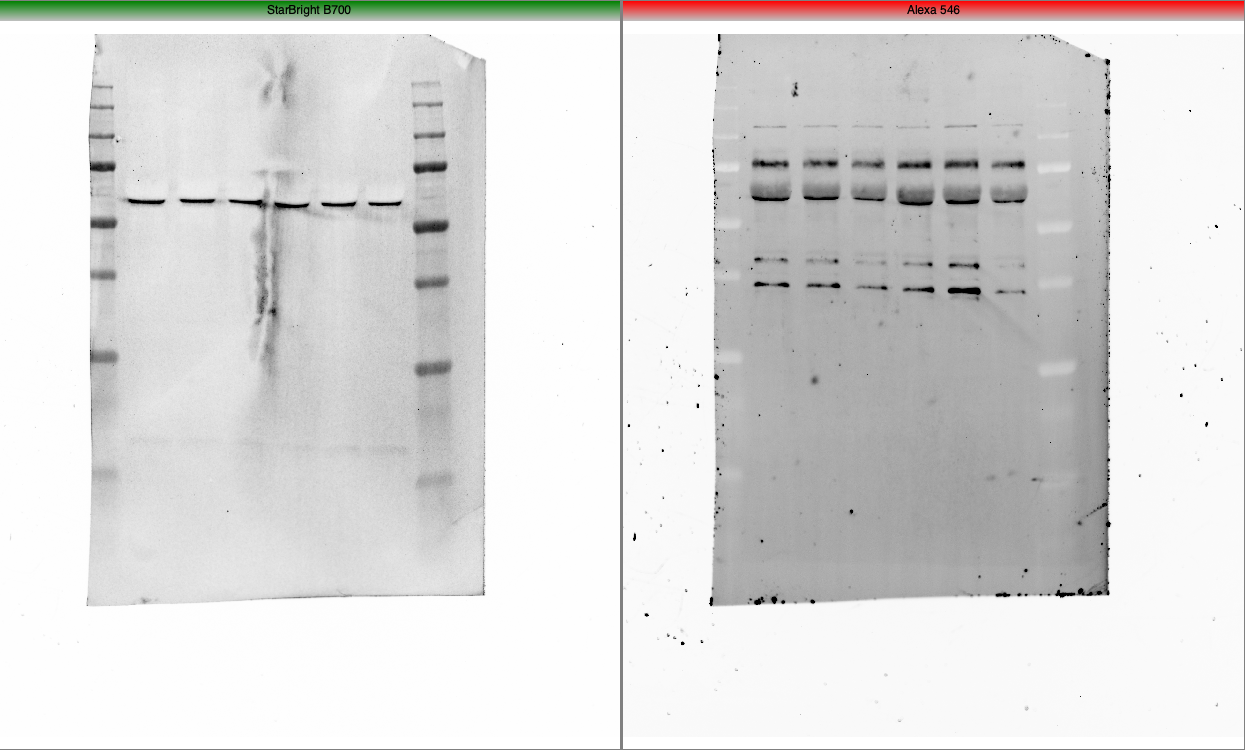


60Kda
AKT


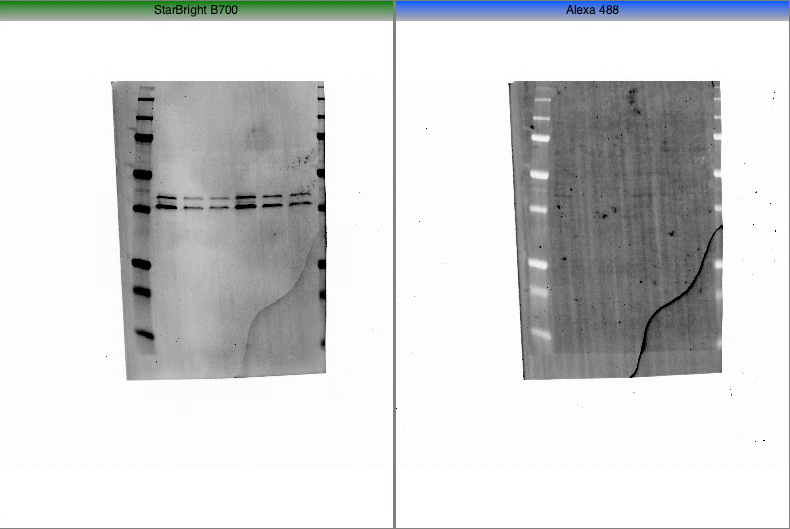


42/44Kda

pERK


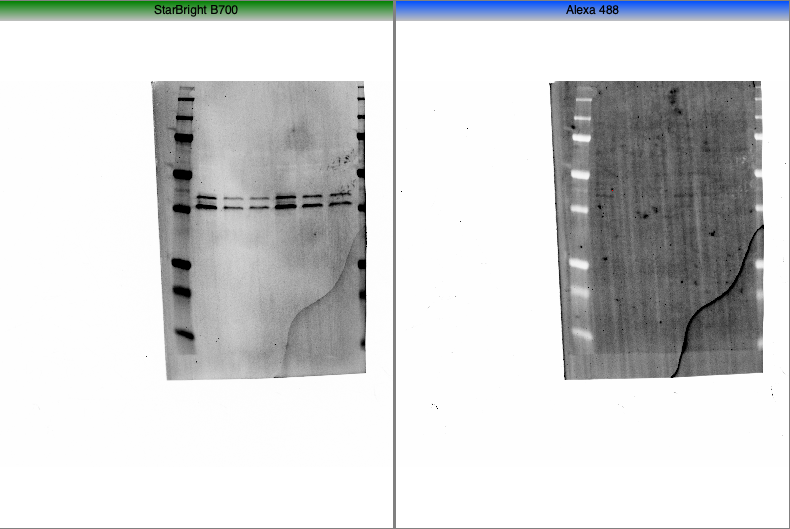


42/44Kda

ERK


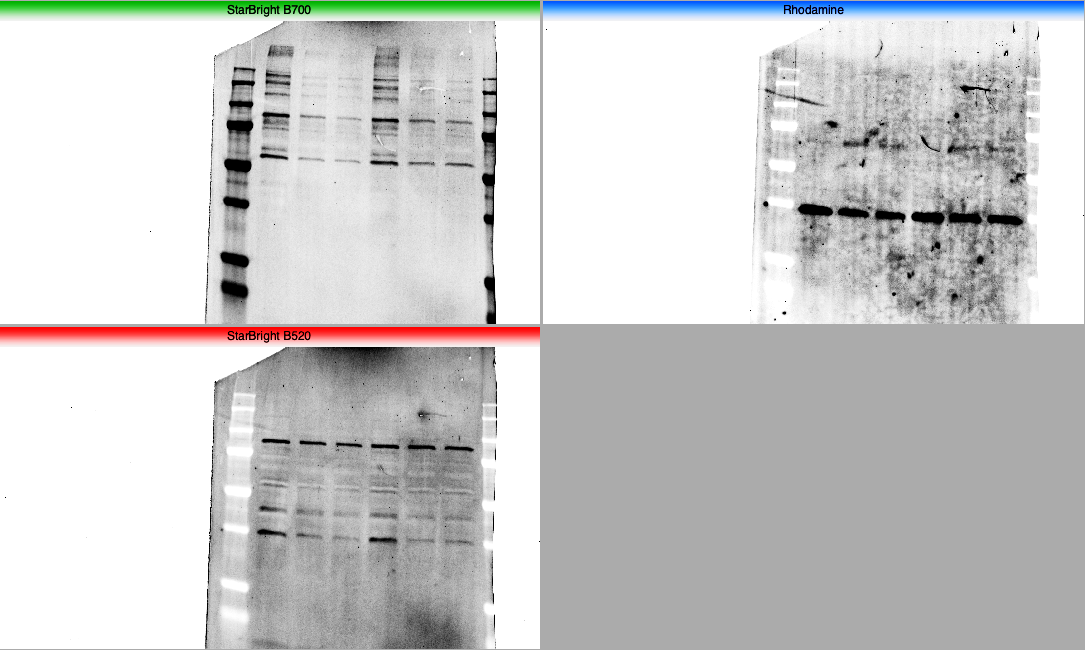


85Kda

pSTAT3


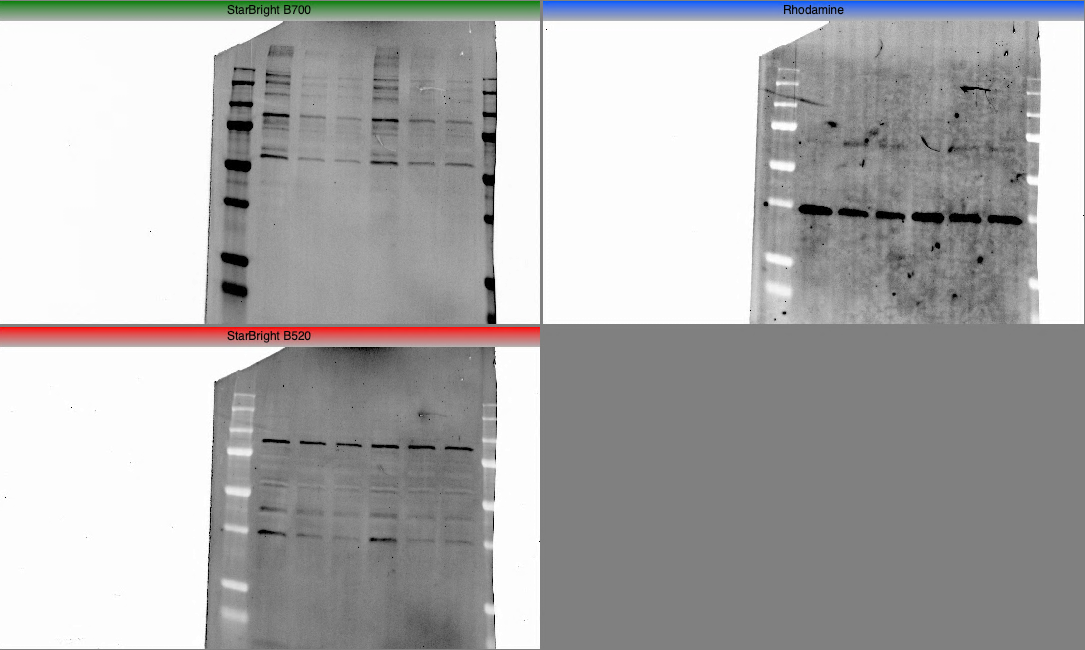


85Kda

STAT3


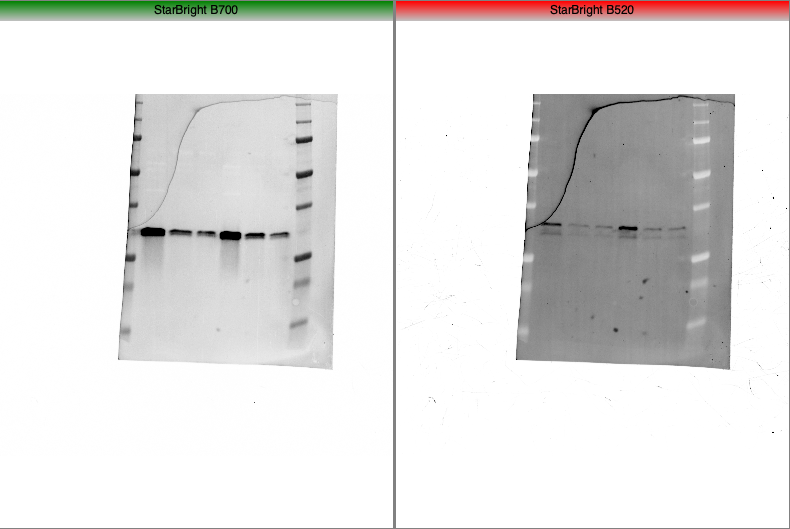


30Kda

pS6


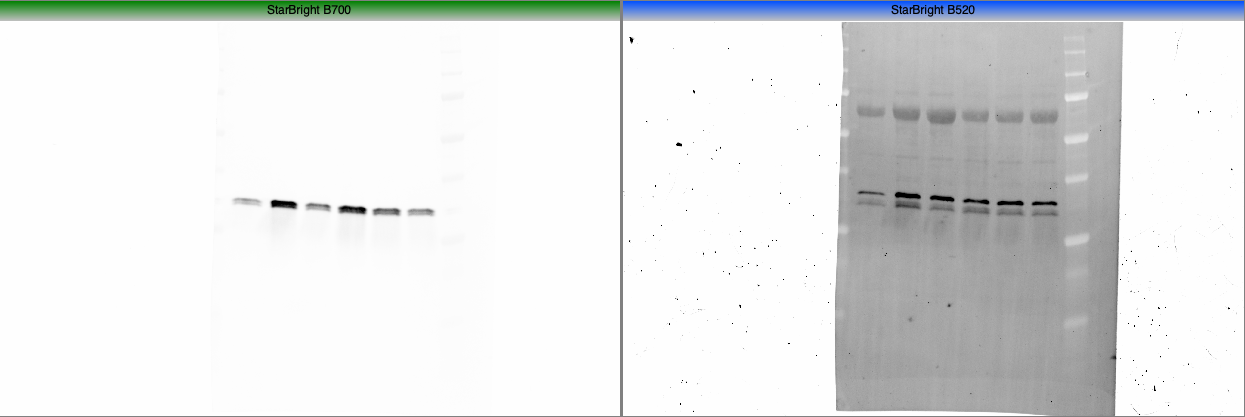


30Kda

S6


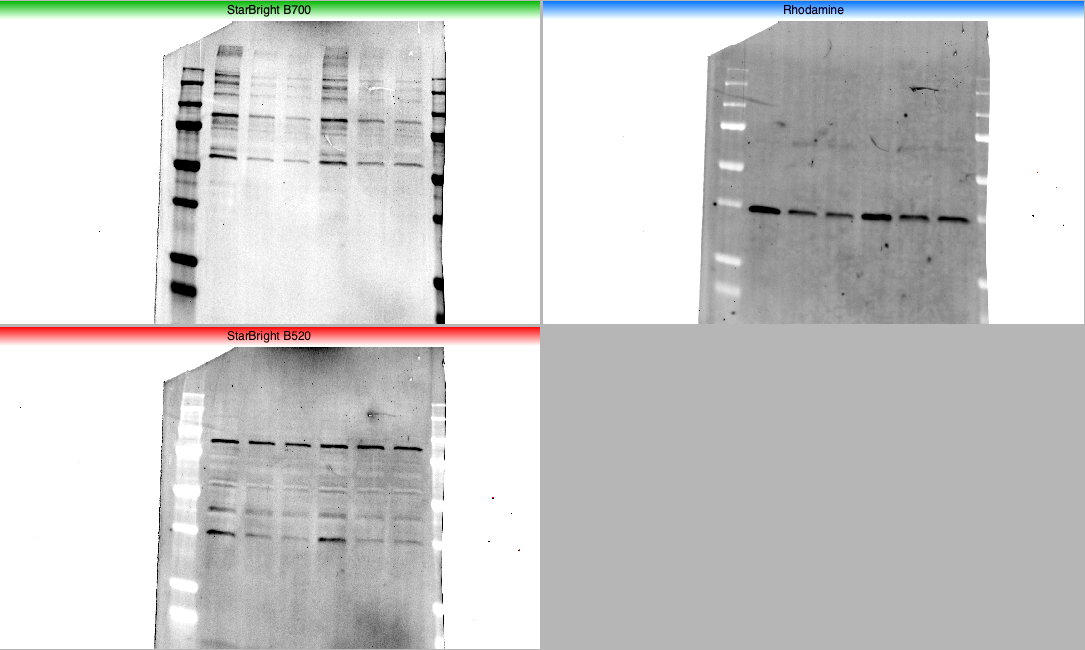


37Kda

GAPDH

CRISPR OP3


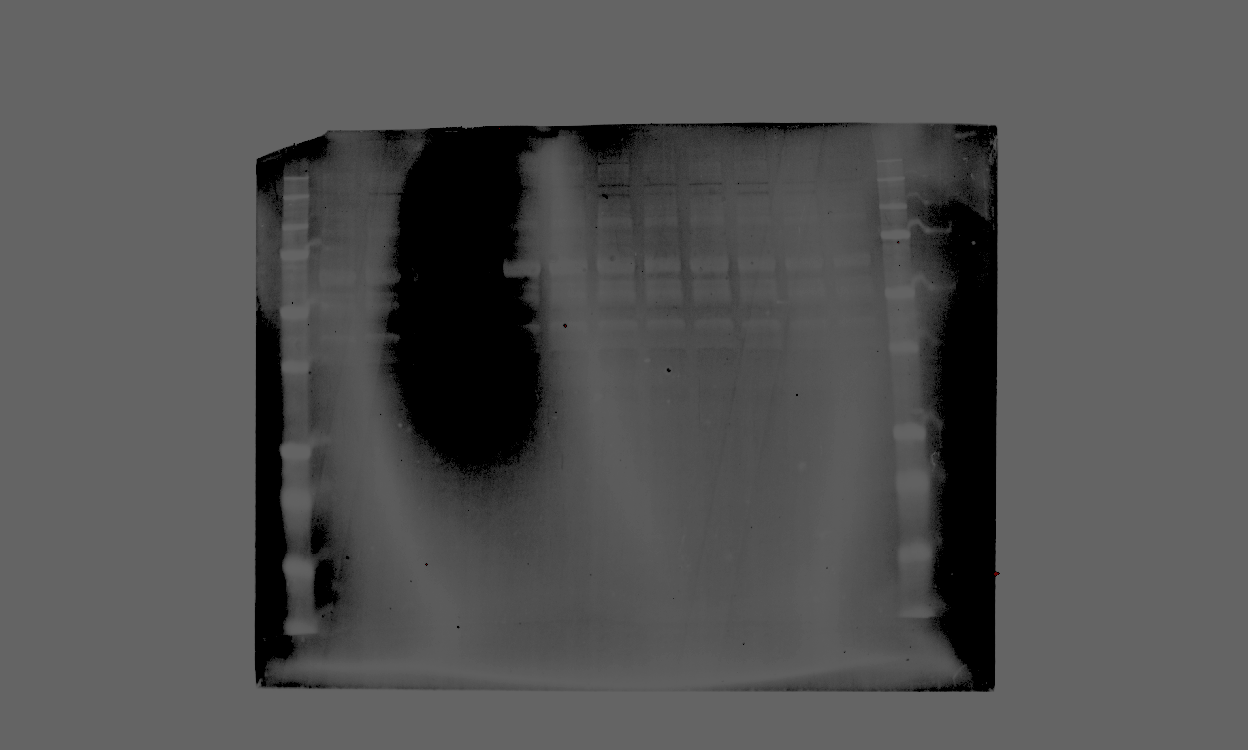


110kda
pTRKb


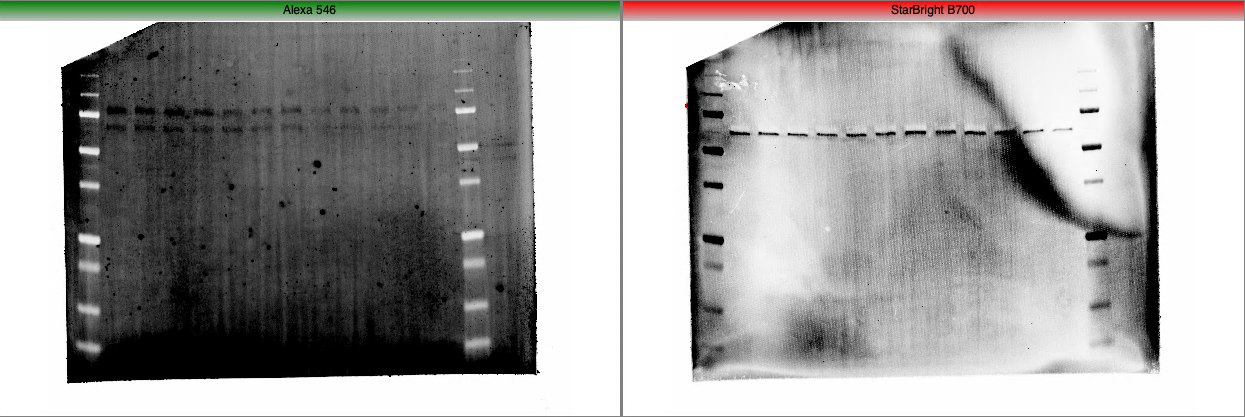


60kDa
pAKT


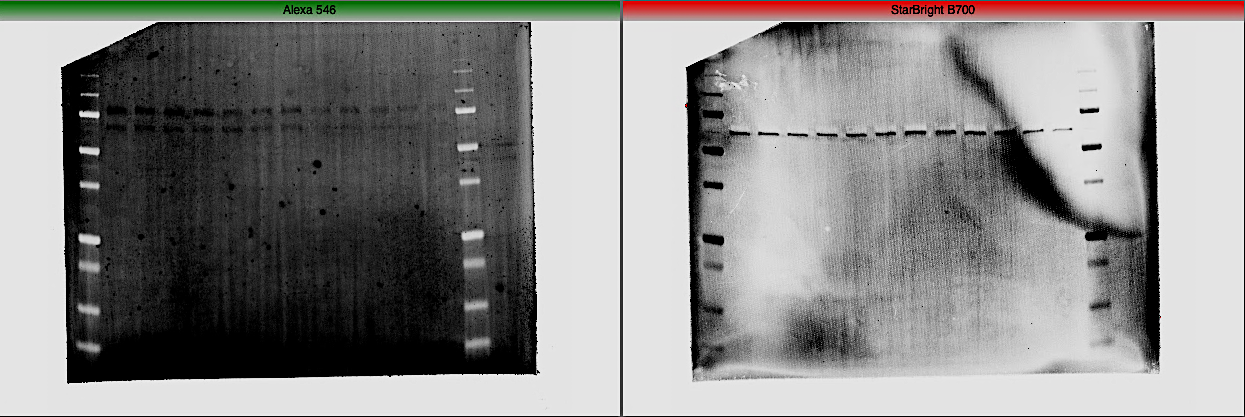


60kda
AKT


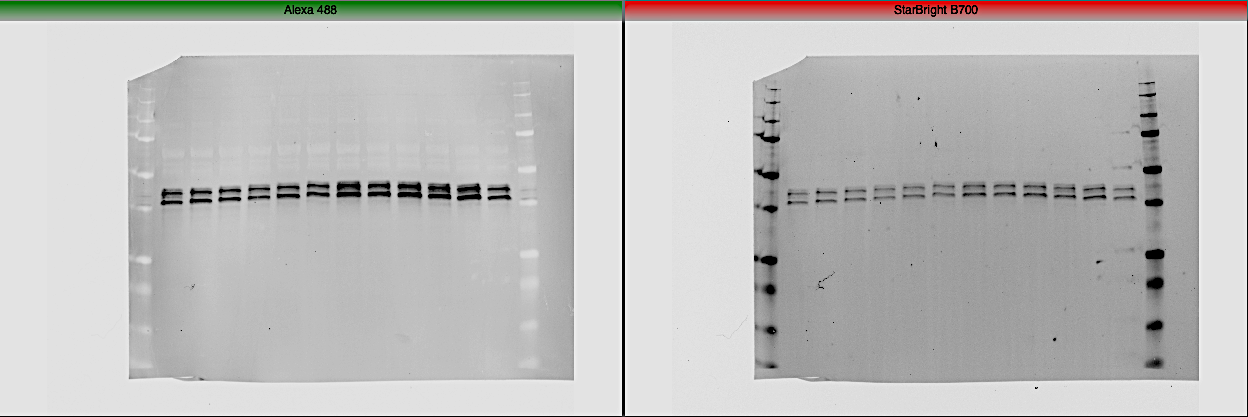


42/44kda

pERK


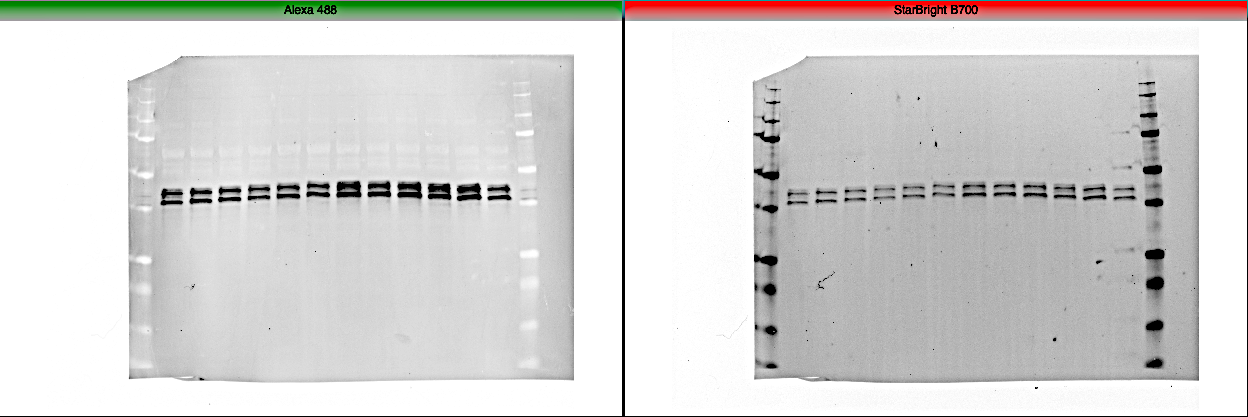


42/44
ERK


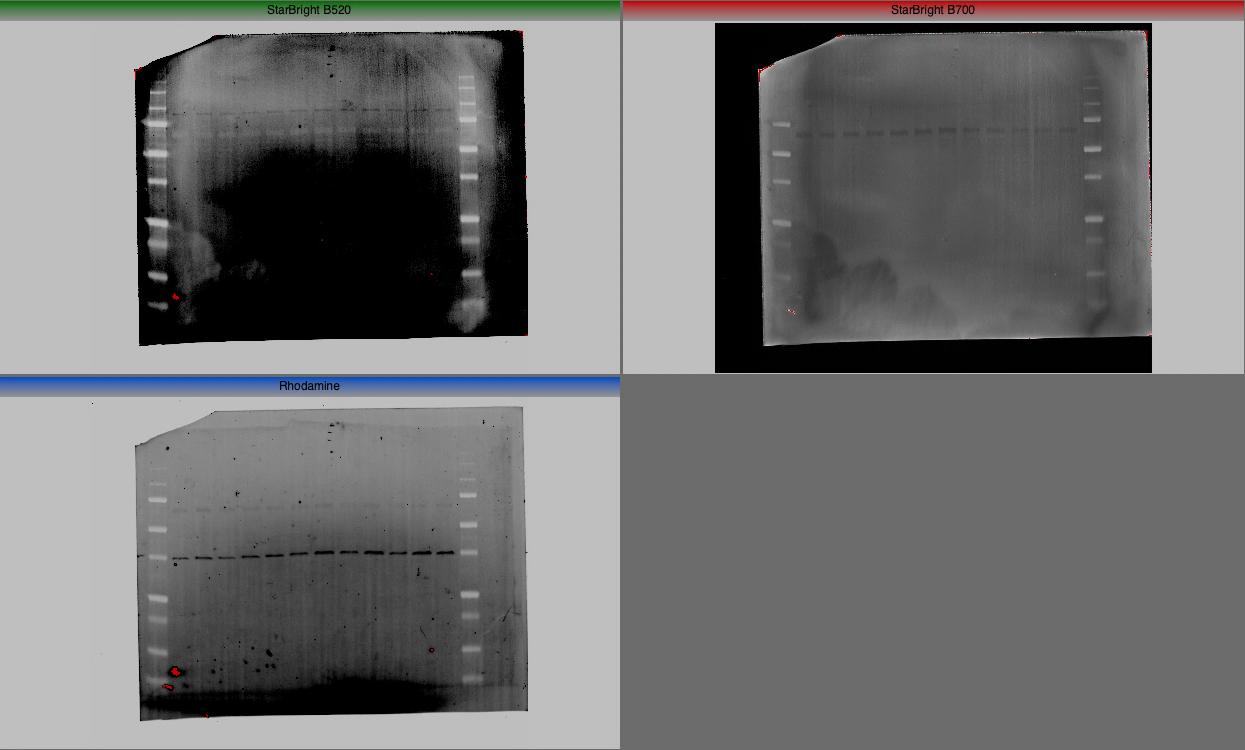


85kda

pSTAT3


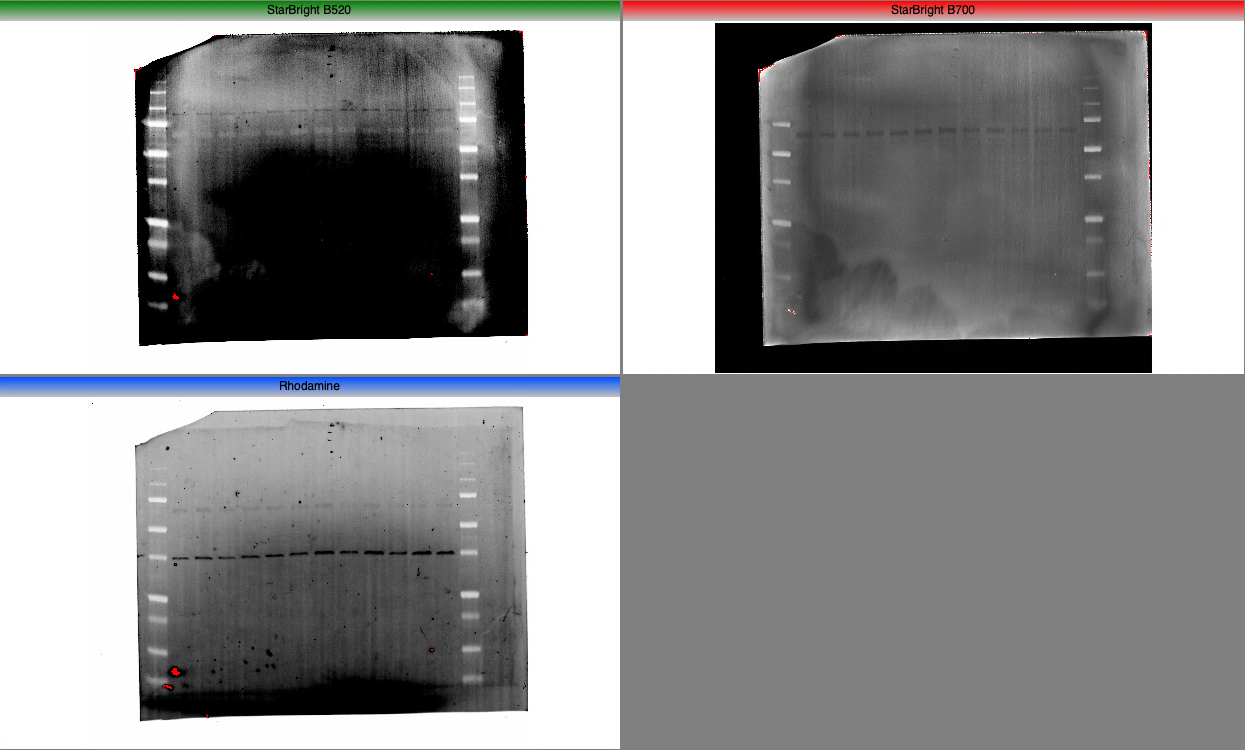


85Kda

STAT3


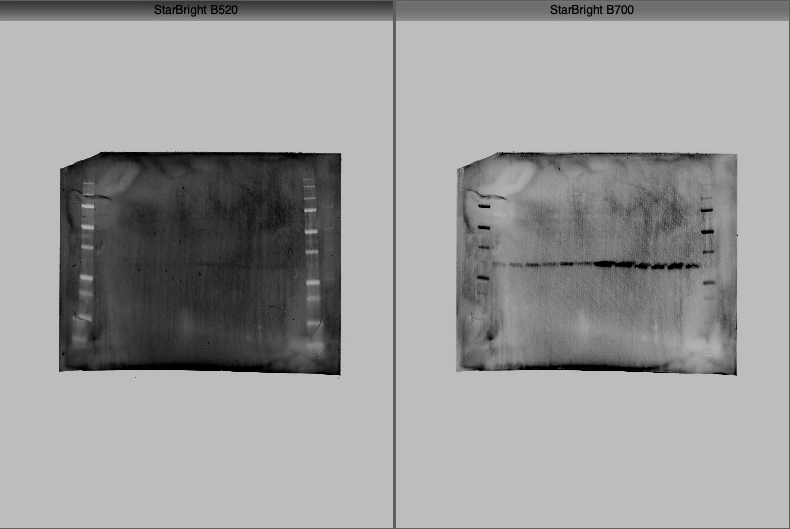


30kda

pS6


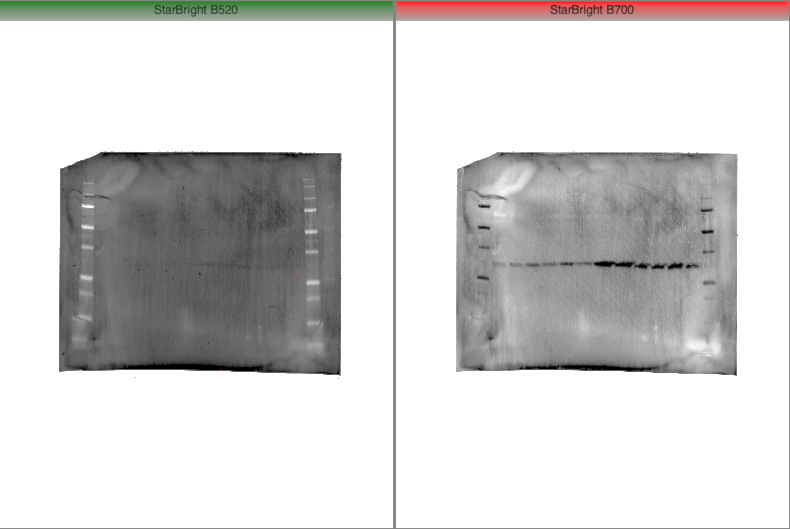


30kda

S6


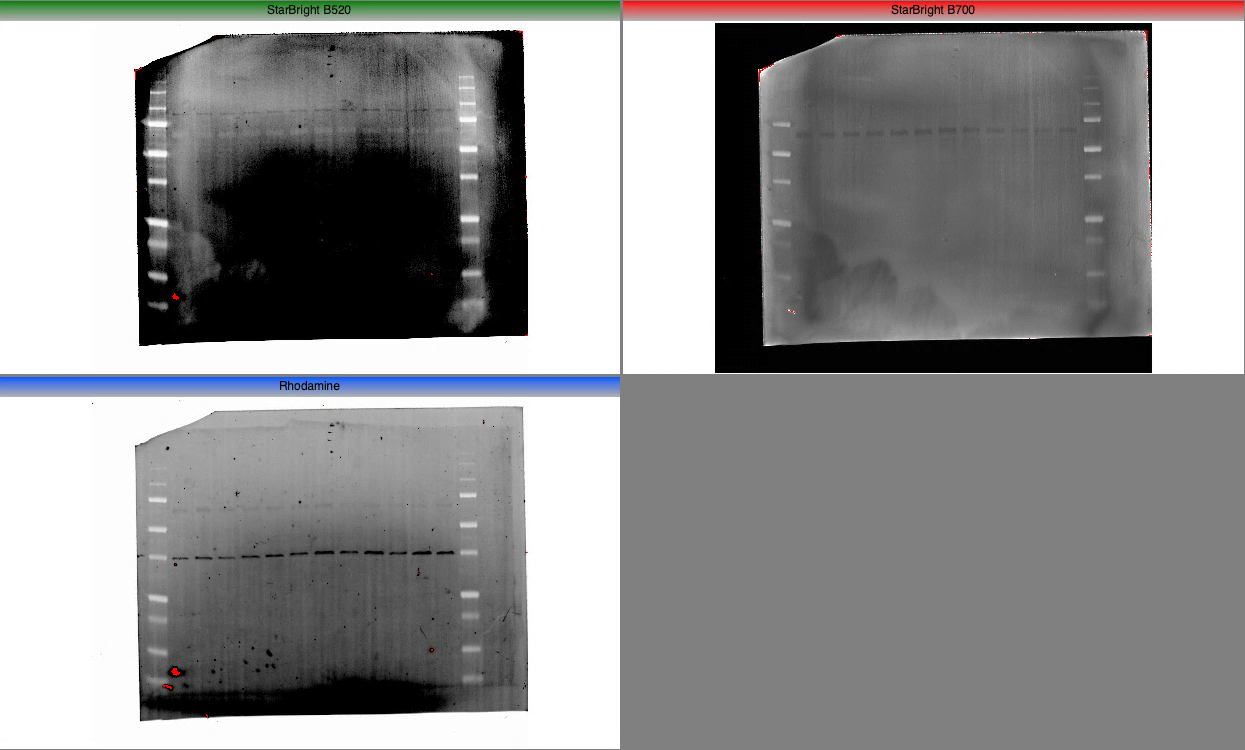


37kDa

GAPDH


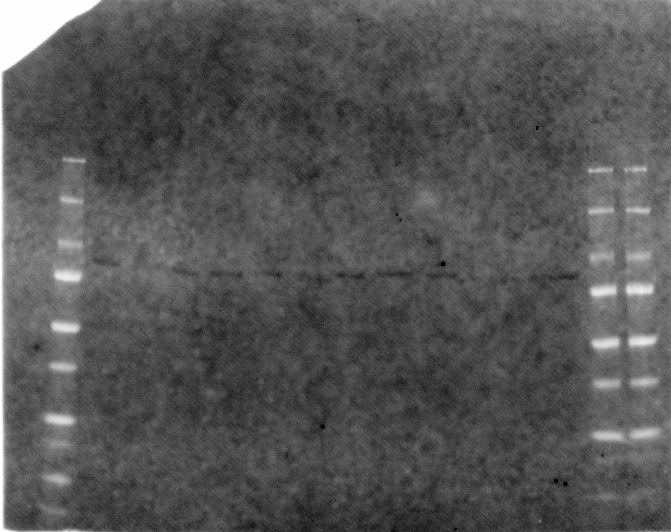
Complement Data

150kda

100kda


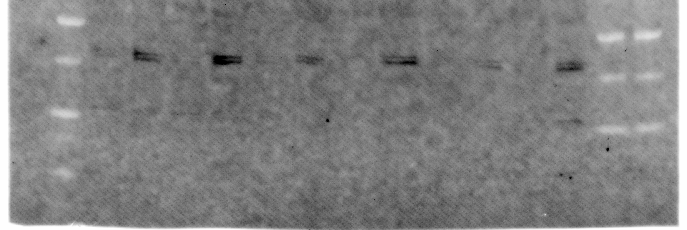
VEGFR

150kda

100kda
C-KIT

Drug OP1

| pAKT  Ser473 | 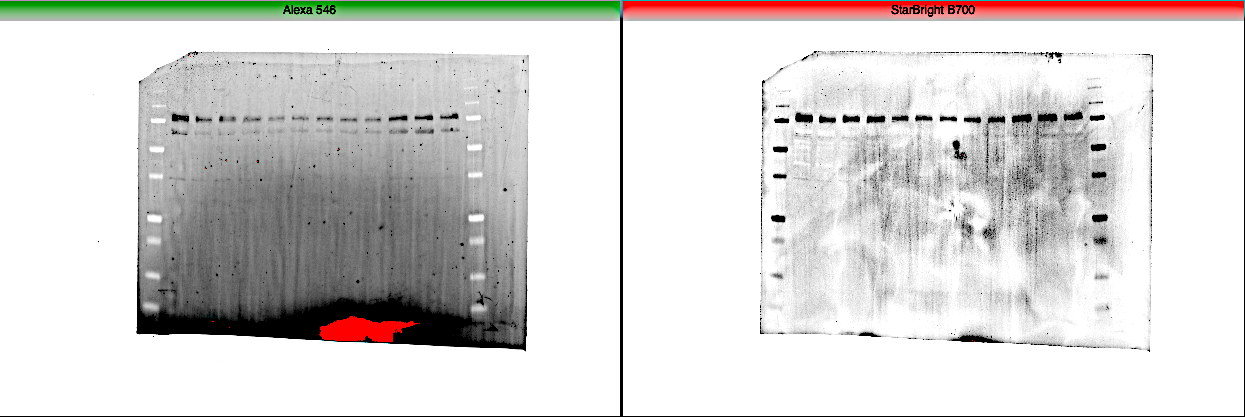 | 60kDa |
| --- | --- | --- |
| AKT | 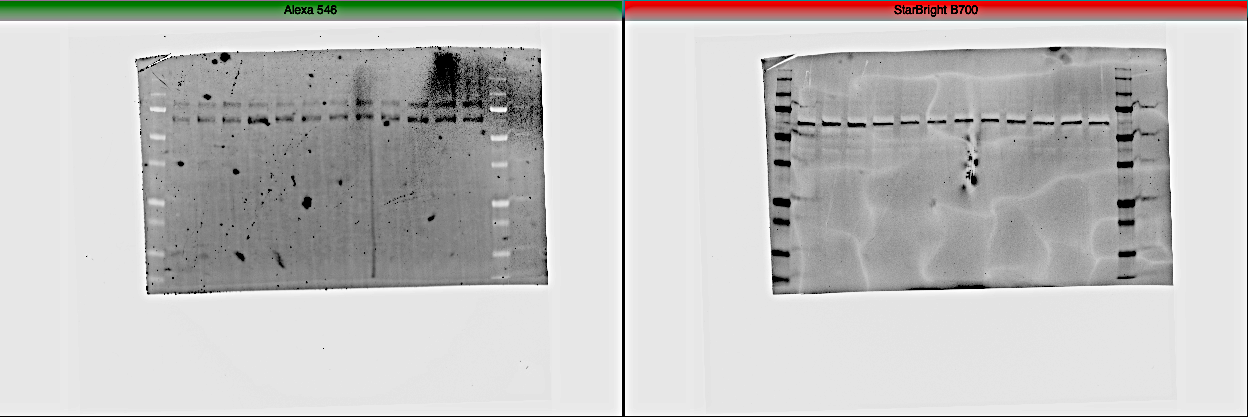 | 60kDa |
| pERK 1/2  Thr202/Tyr204 | 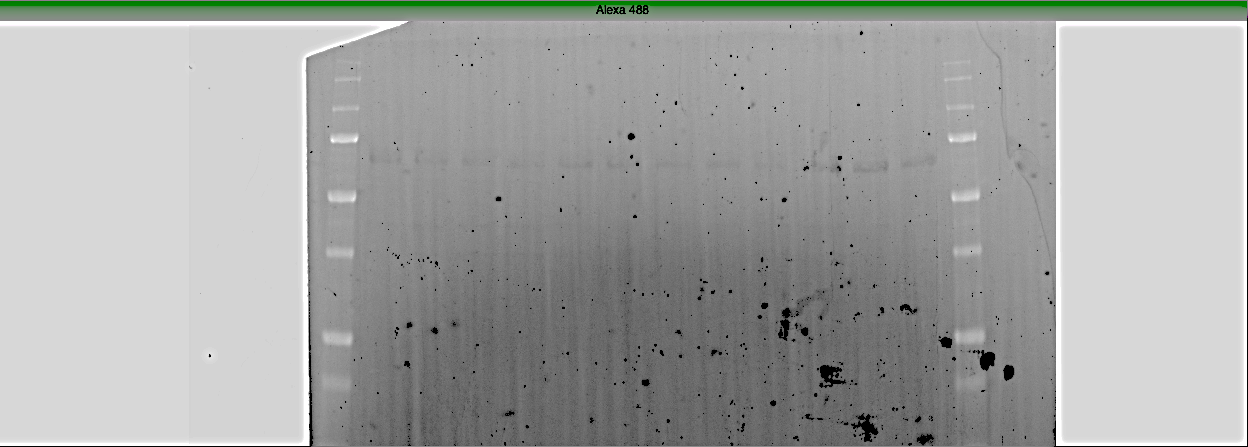 | 42/44kDa |
| ERK 1/2 | 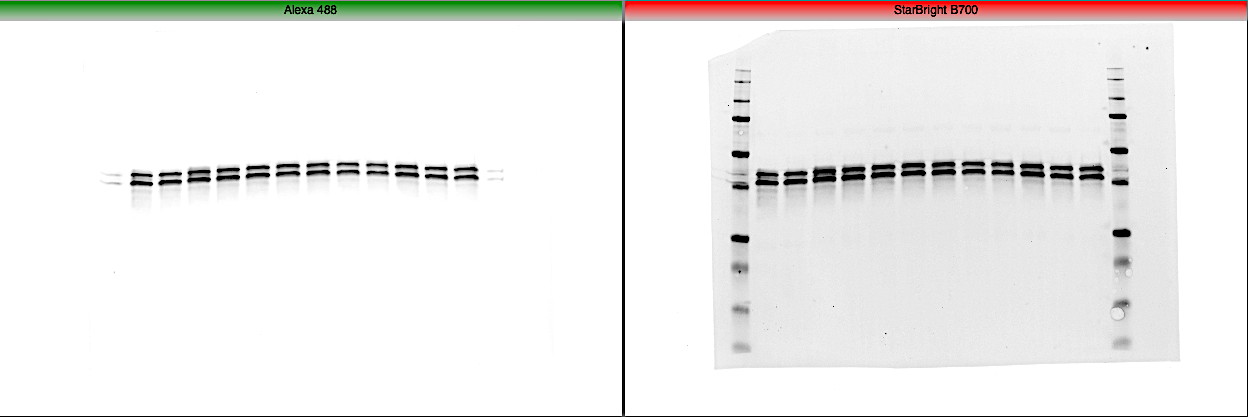 | 42/44kDa |
| pSTAT3  Ser727 | 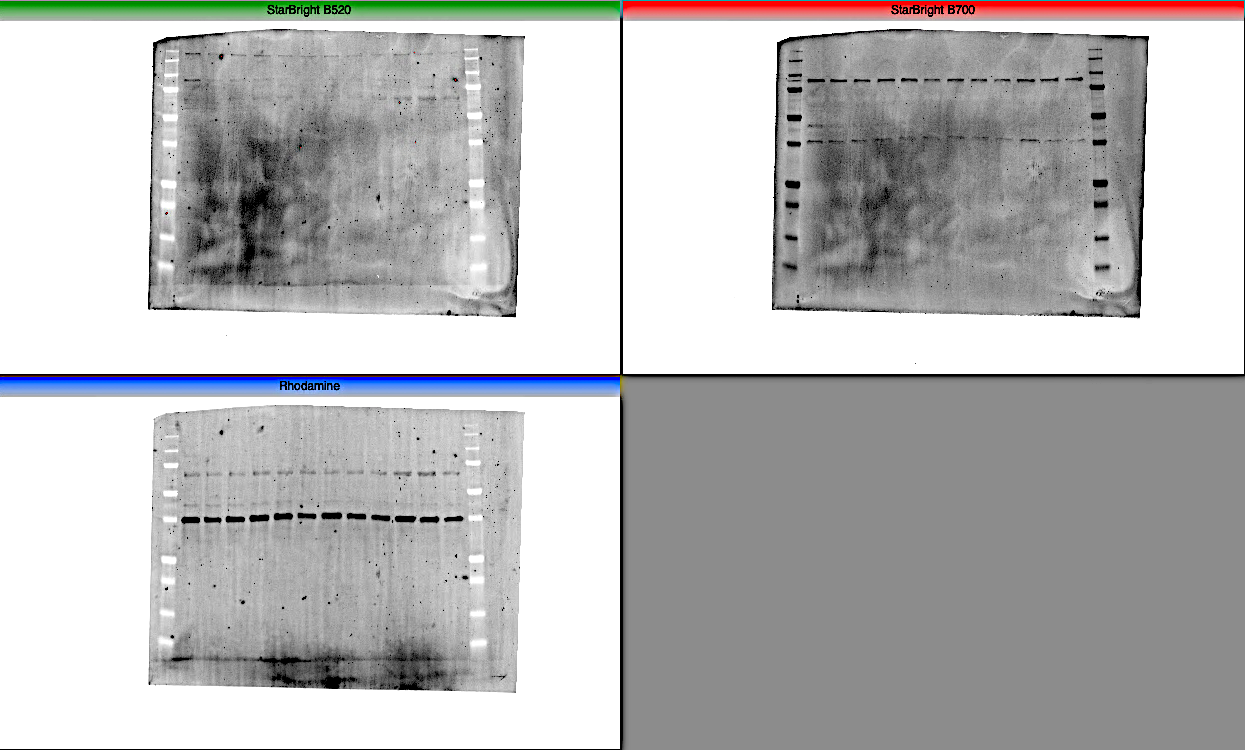 | 85kDa |
| STAT3 | 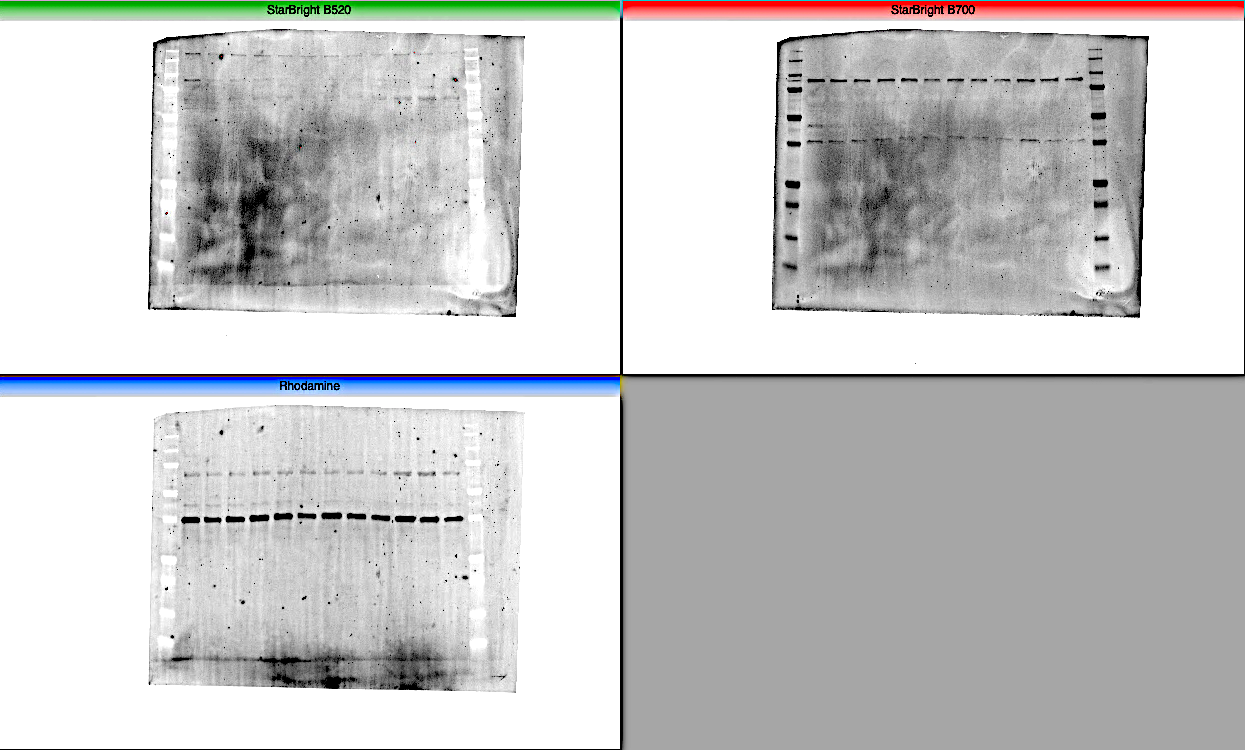 | 85kDa |
| pS6  Ser235/236 | 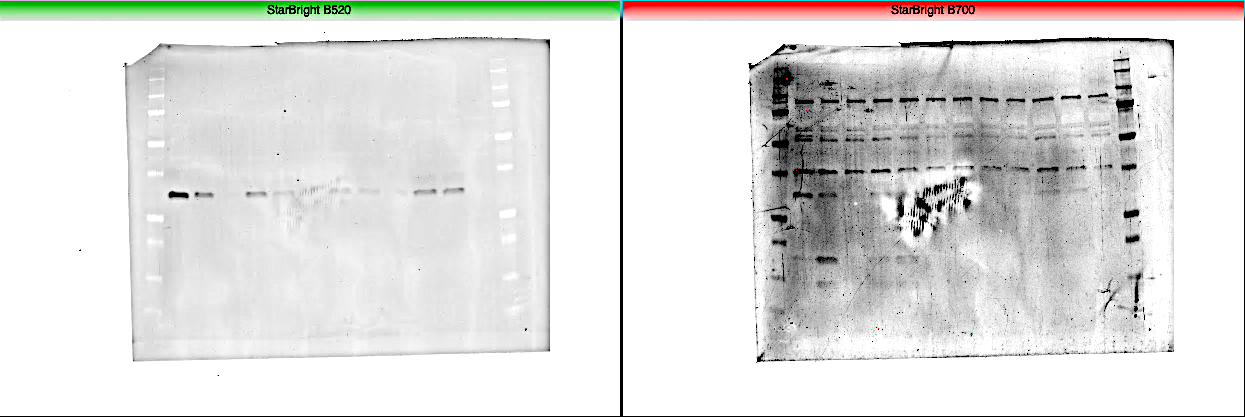 | 30kDa |
| S6 | 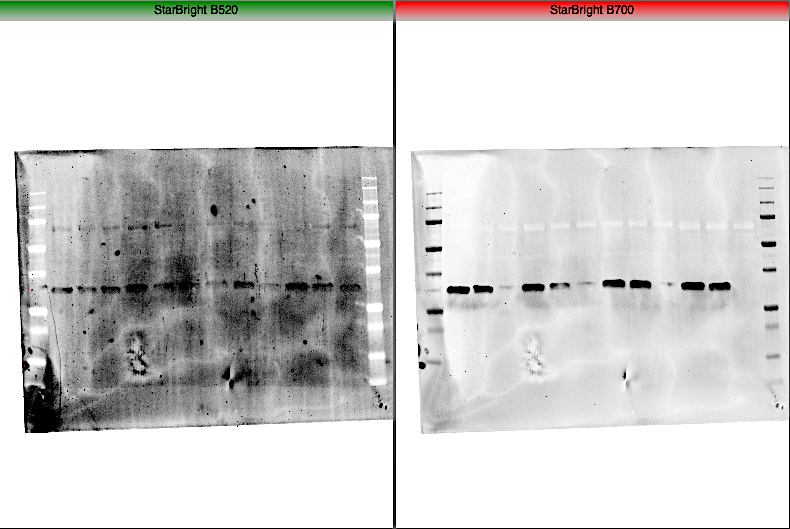 | 30kDa |
| GAPDH | 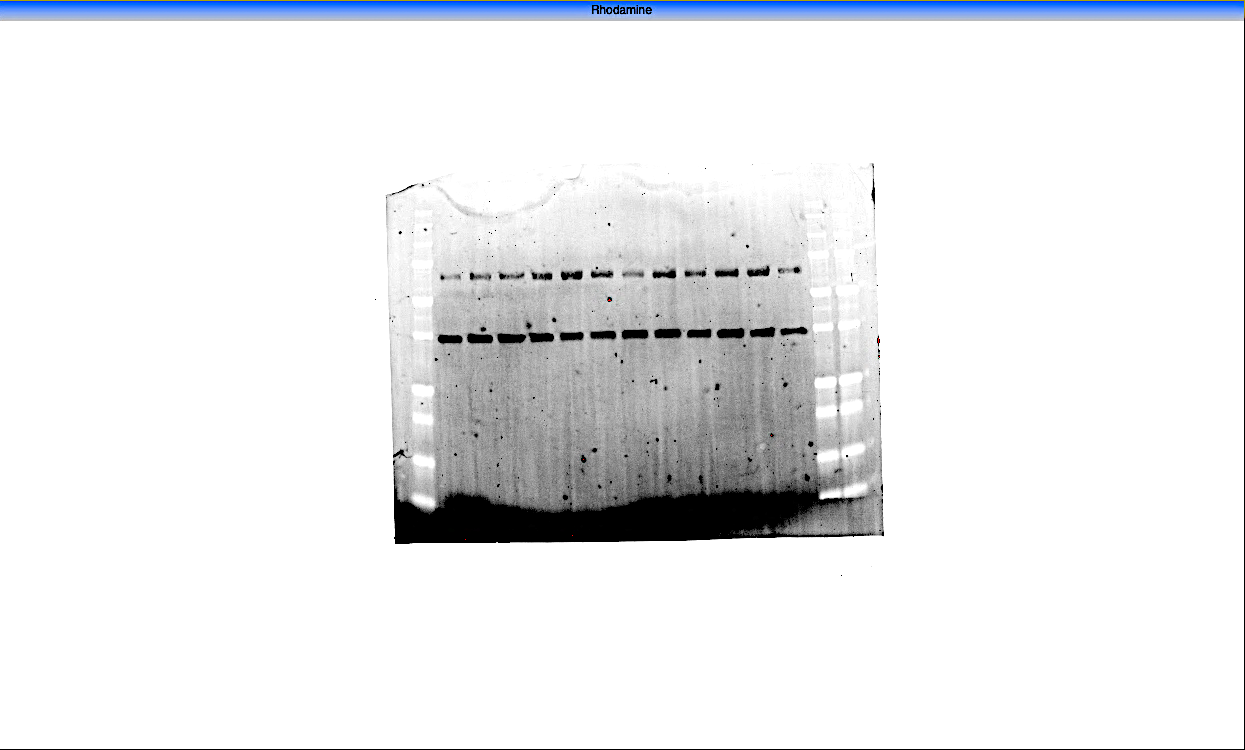 | 37kDa |

OP3

| pAKT  Ser473 | 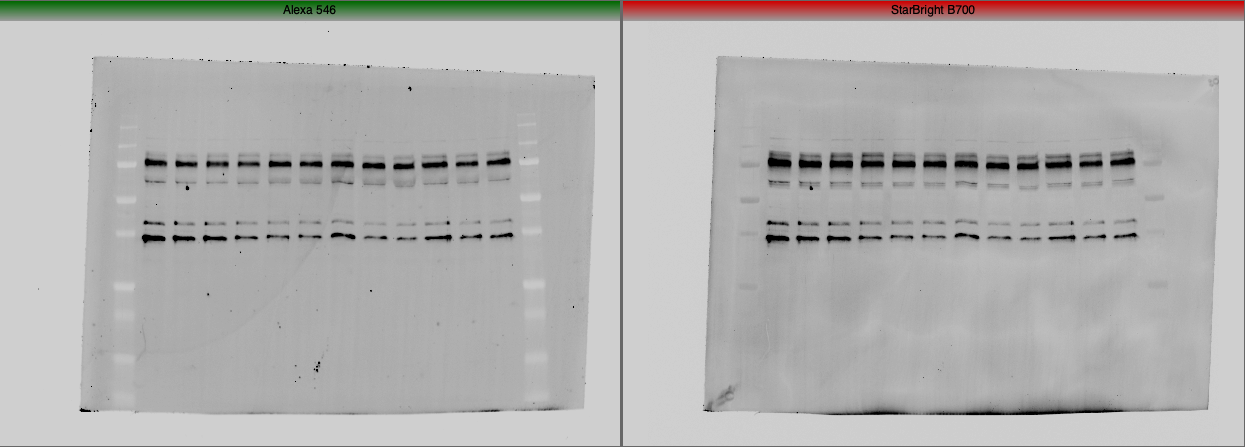 | 60kDa |
| --- | --- | --- |
| AKT | 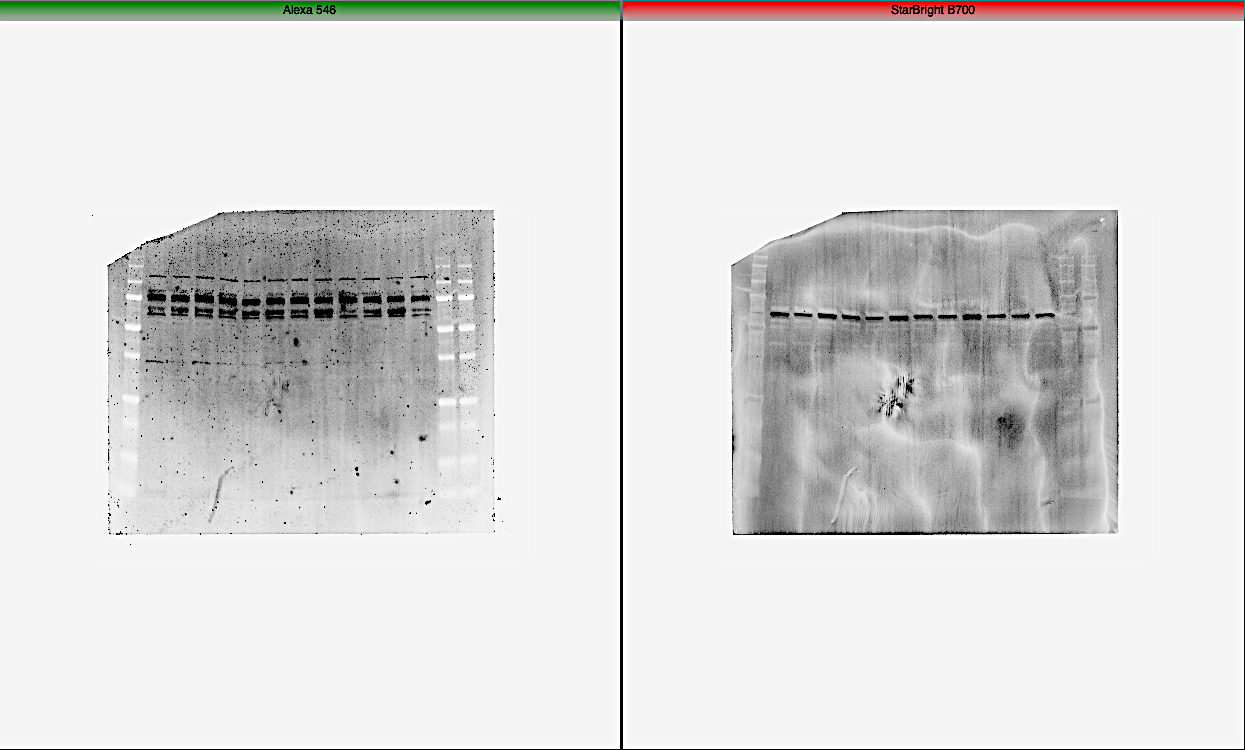 | 60kDa |
| pERK 1/2  Thr202/Tyr204 | 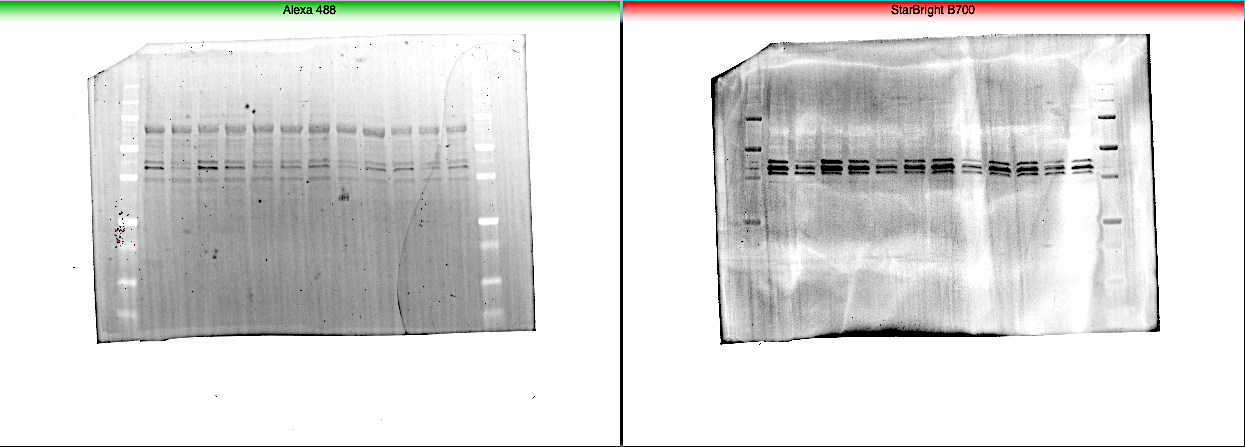 | 42/44kDa |
| ERK 1/2 | 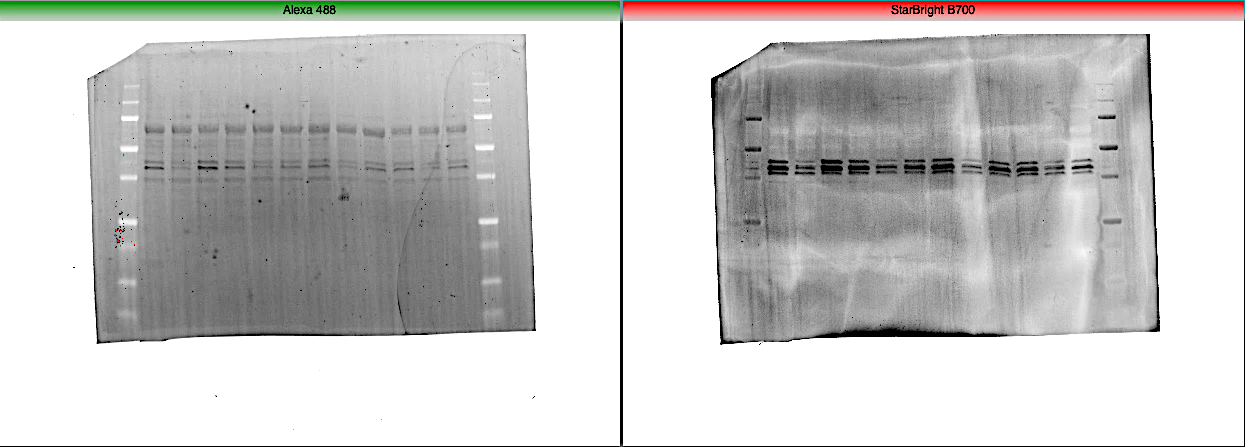 | 42/44kDa |
| pSTAT3  Ser727 | 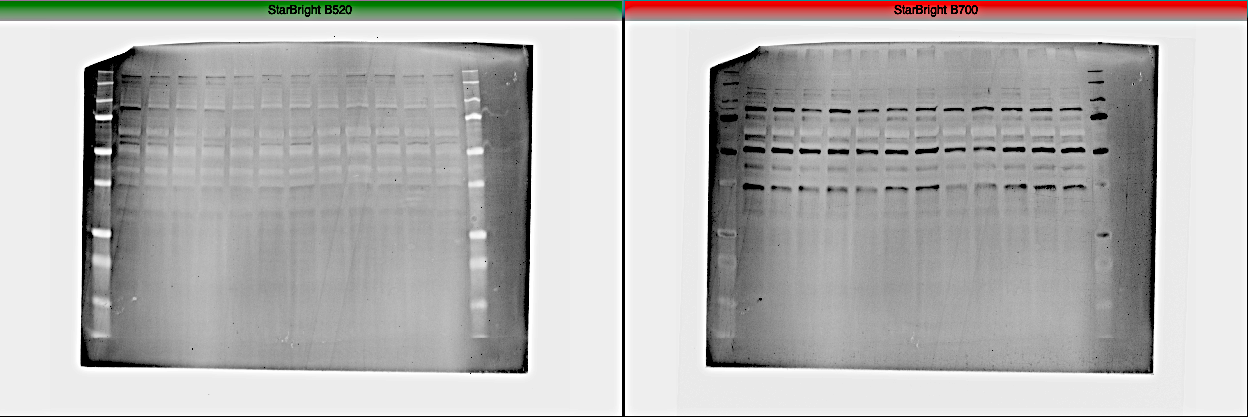 | 85kDa |
| STAT3 | 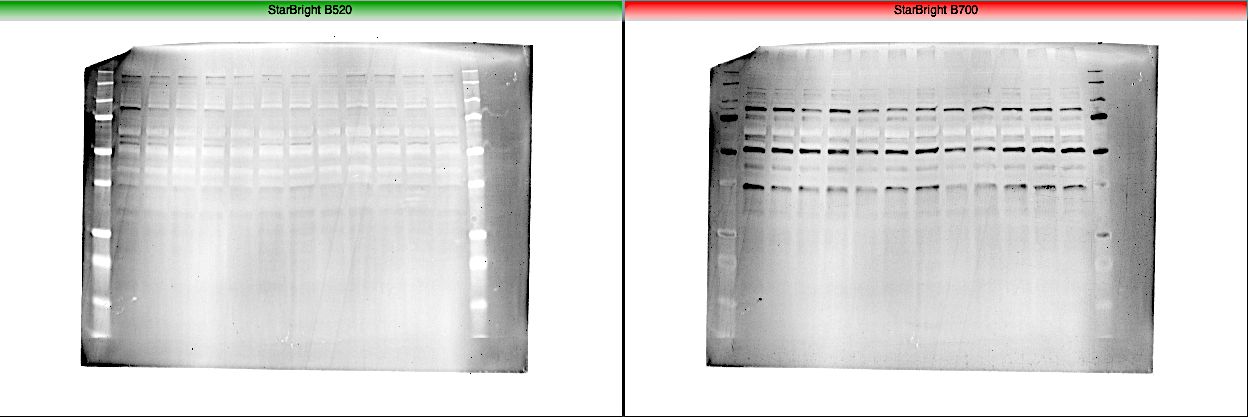 | 85kDa |
| pS6  Ser235/236 | 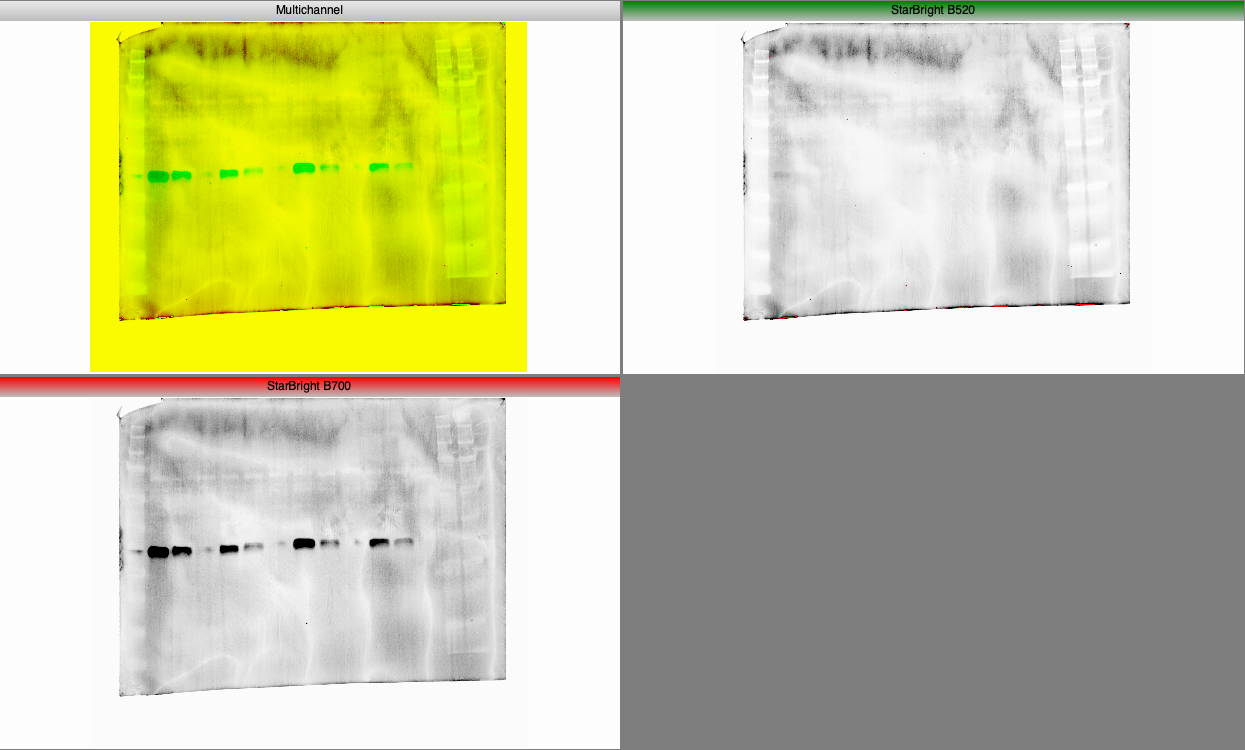 | 30kDa |
| S6 | 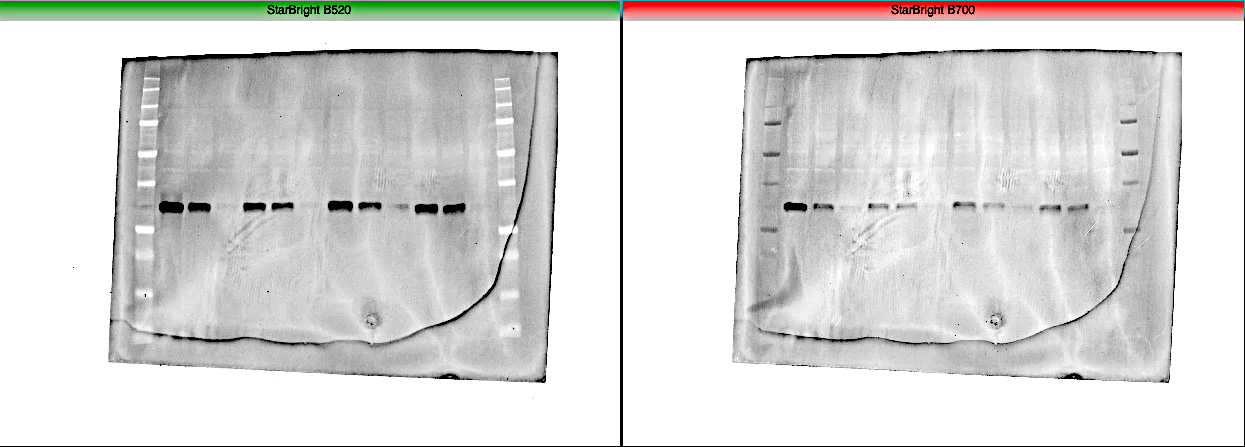 | 30kDa |
| GAPDH | 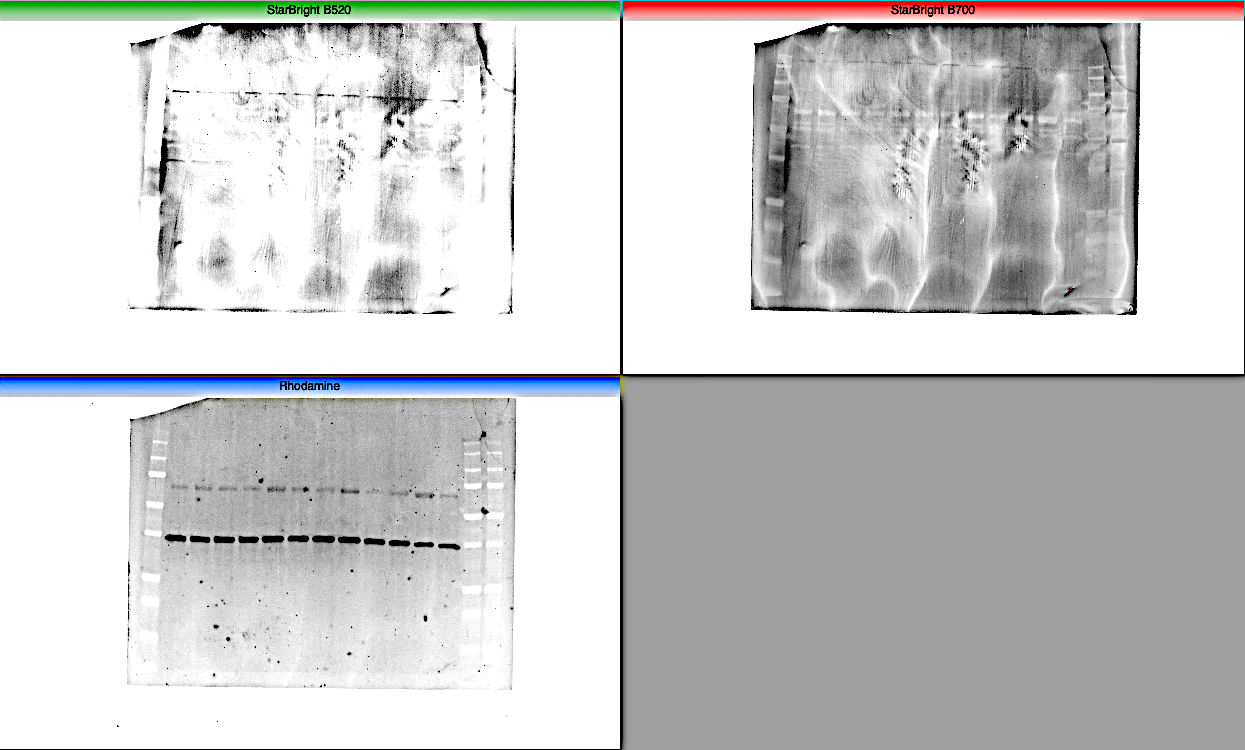 | 37kDa |

HEK

| Flag | 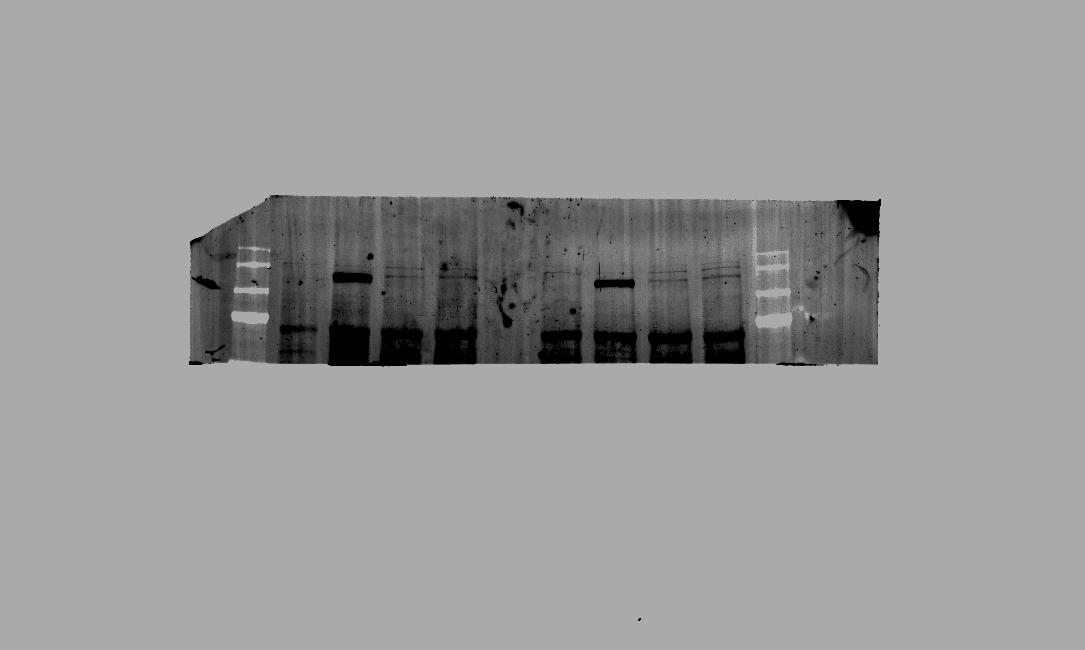 | 110kDa |
| --- | --- | --- |
| pTRKb  Tyr 516 | 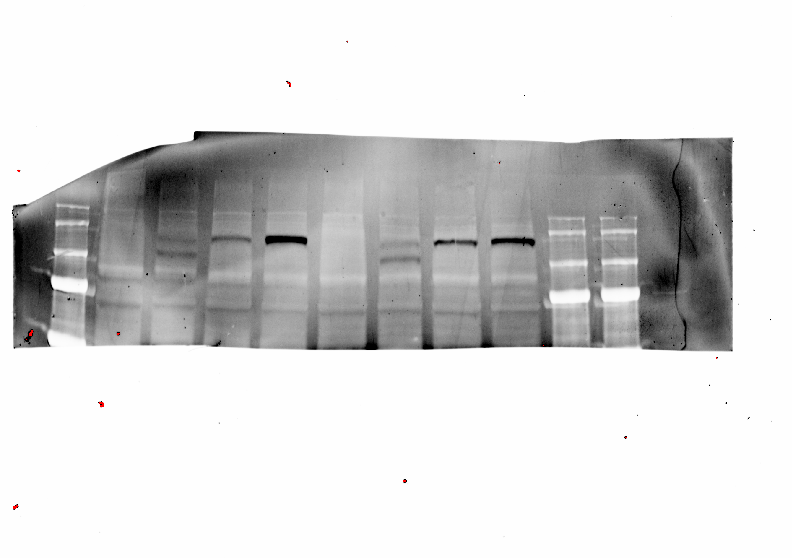 | 110kDa |
| pAKT  Ser473 | 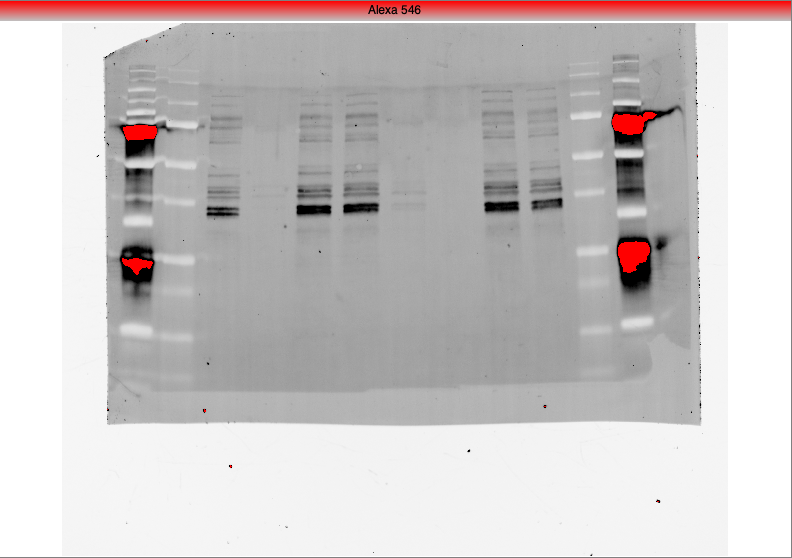 | 60kDa |
| AKT | 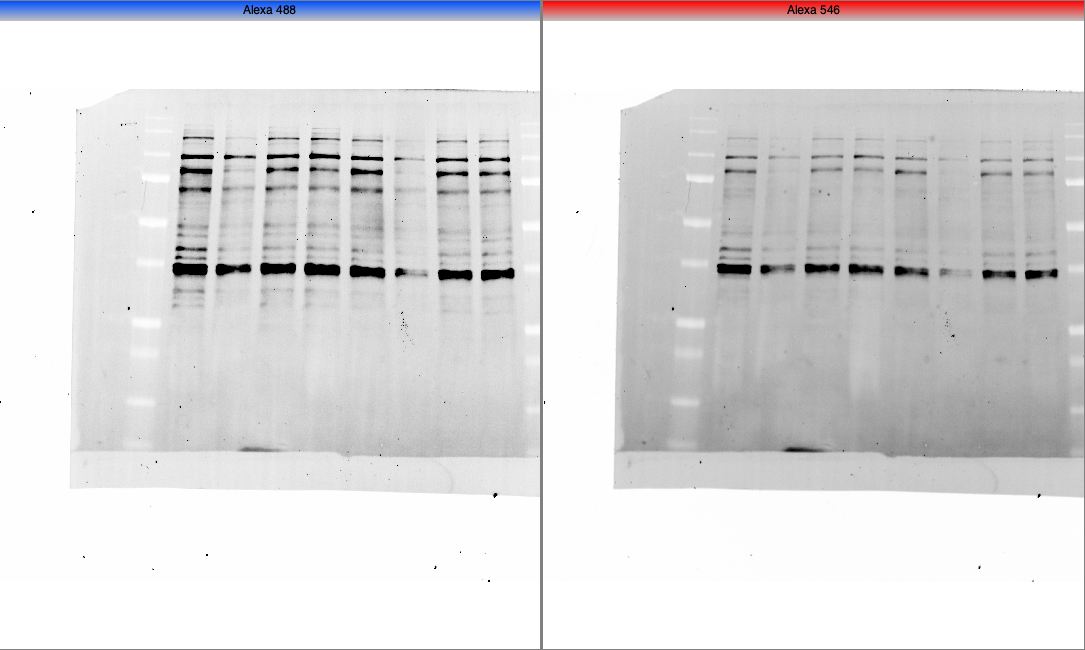 | 60kDa |
| pERK 1/2  Thr202/Tyr204 | 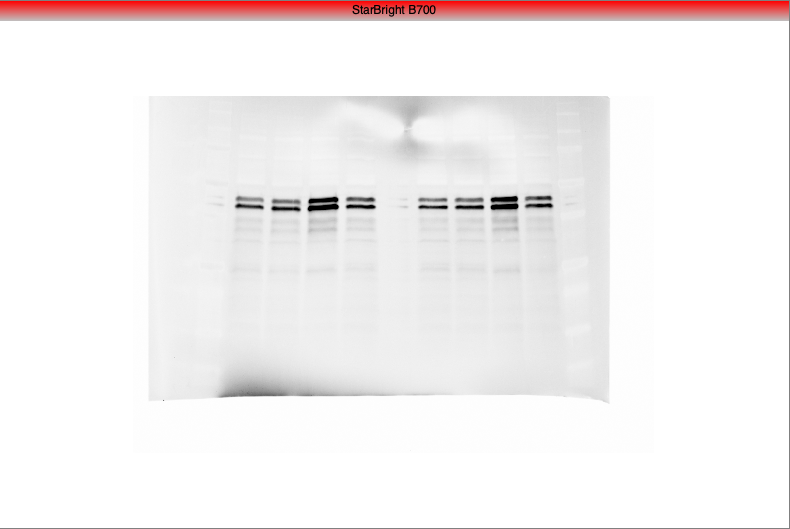 | 42/44kDa |
| ERK 1/2 | 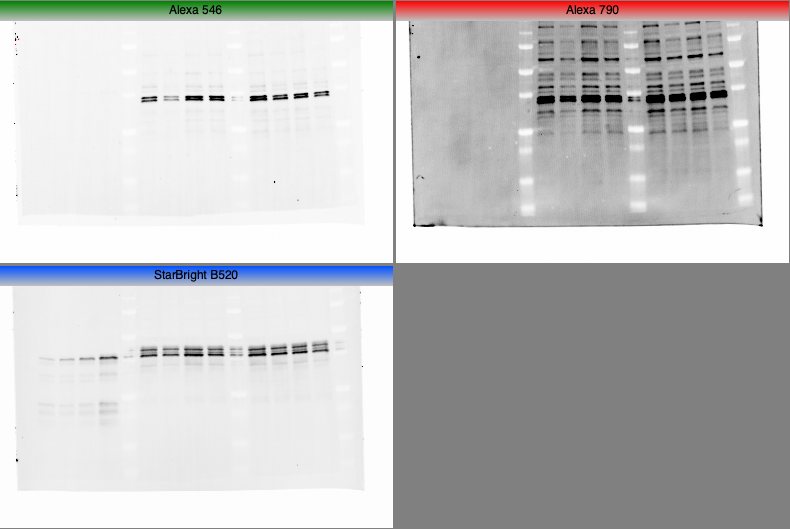 | 42/44kDa |
| pSTAT3  Ser727 | 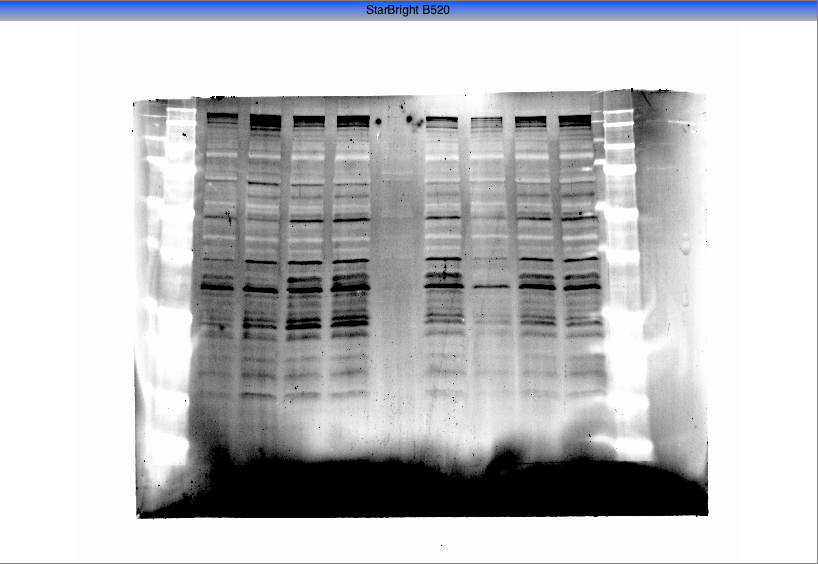 | 85kDa |
| STAT3 | 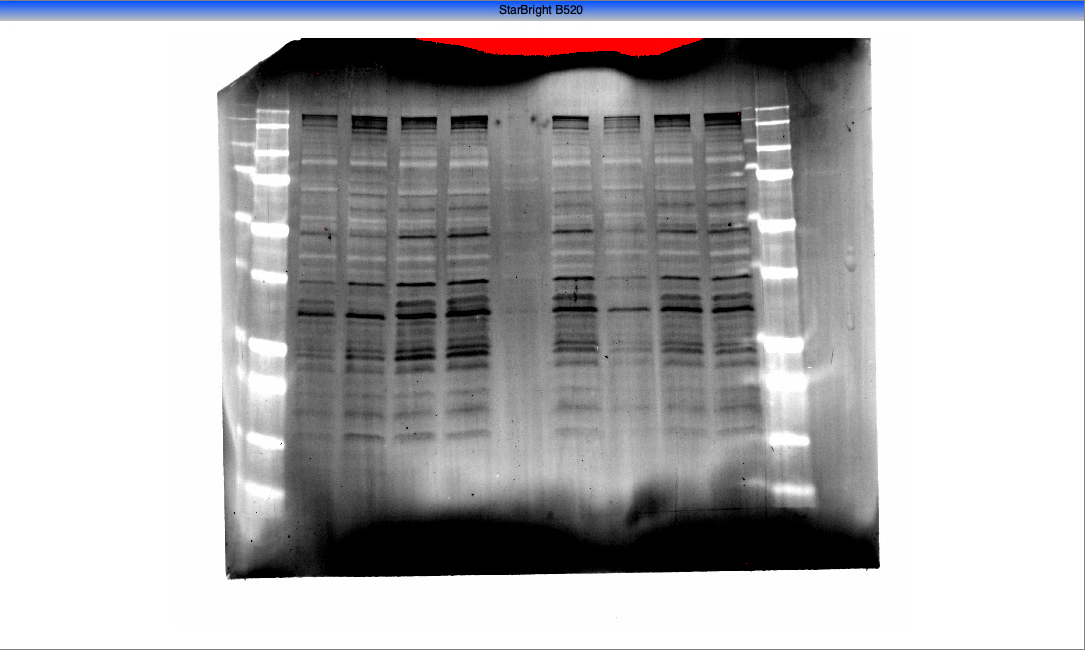 | 85kDa |
| pS6  Ser235/236 | 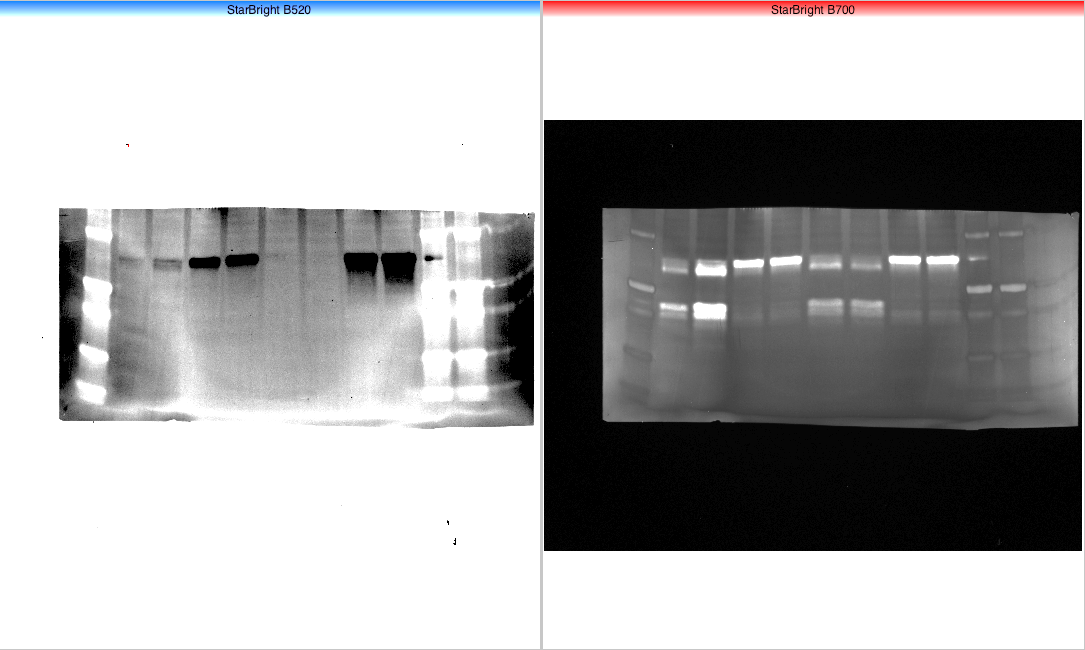 | 30kDa |
| S6 | 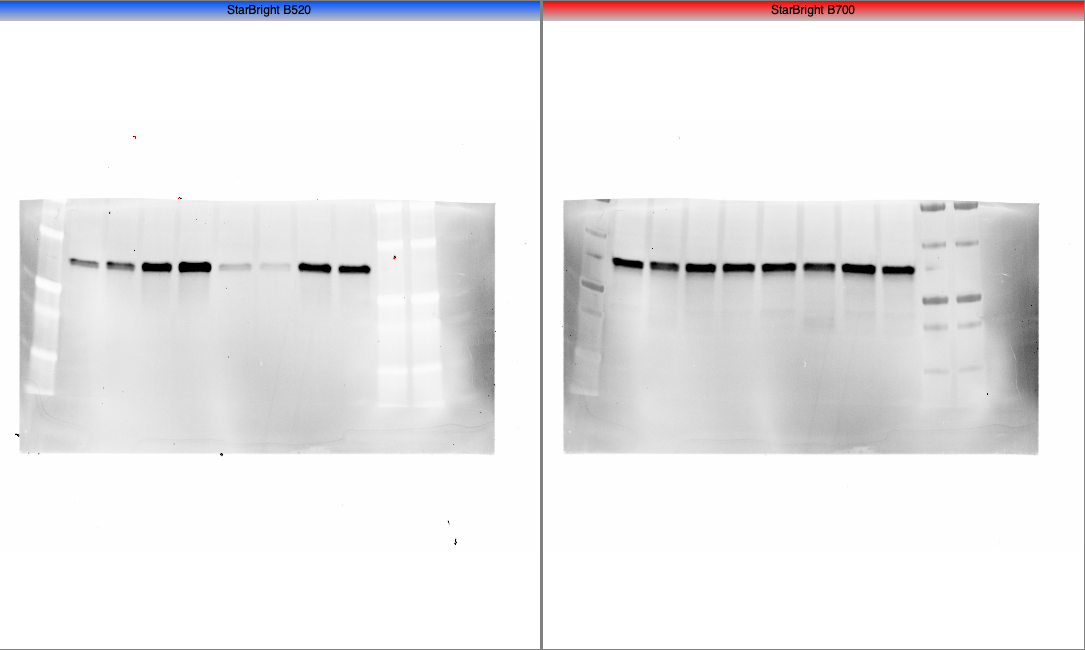 | 30kDa |
| GAPDH | 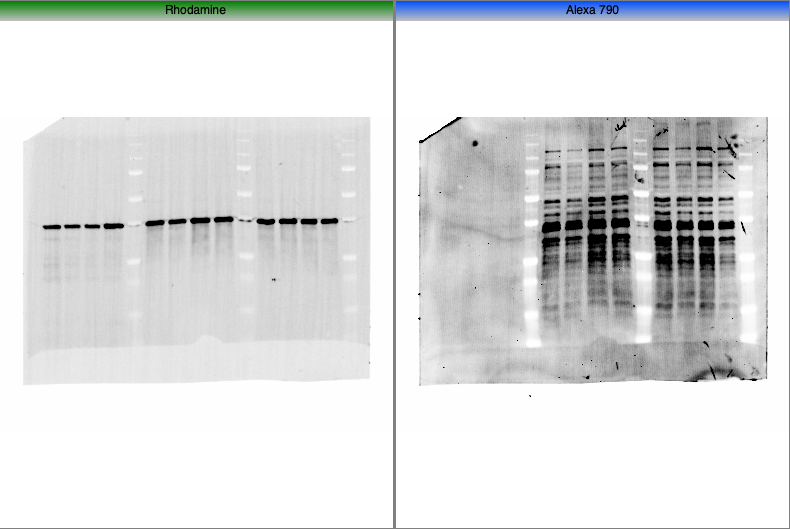 | 37kDa |
